# Tantalum–Zirconium Co‐Doped Metal–Organic Frameworks Sequentially Sensitize Radio–Radiodynamic–Immunotherapy for Metastatic Osteosarcoma

**DOI:** 10.1002/advs.202206779

**Published:** 2023-02-05

**Authors:** Tao Li, Mingquan Gao, Zifei Wu, Junjun Yang, Banghui Mo, Songtao Yu, Xiaoyuan Gong, Jing Liu, Weidong Wang, Shenglin Luo, Rong Li

**Affiliations:** ^1^ Institute of Combined Injury State Key Laboratory of Trauma Burns and Combined Injury Chongqing Engineering Research Center for Nanomedicine College of Preventive Medicine Third Military Medical University (Army Medical University) Chongqing 400038 China; ^2^ Center for Joint Surgery Southwest Hospital Third Military Medical University (Army Medical University) Chongqing 400038 China; ^3^ Department of Radiation Oncology Sichuan Cancer Hospital & Institute Sichuan Key Laboratory of Radiation Oncology Chengdu Sichuan 610041 China; ^4^ Department of Oncology Southwest Hospital Third Military Medical University (Army Medical University) Chongqing 400038 China

**Keywords:** cancer theranostics, immunotherapy, metal–organic frameworks, radiodynamic therapy, radiosensitizers

## Abstract

Due to radiation resistance and the immunosuppressive microenvironment of metastatic osteosarcoma, novel radiosensitizers that can sensitize radiotherapy (RT) and antitumor immunity synchronously urgently needed. Here, the authors developed a nanoscale metal–organic framework (MOF, named TZM) by co‐doping high‐atomic elements Ta and Zr as metal nodes and porphyrinic molecules (tetrakis(4‐carboxyphenyl)porphyrin (TCPP)) as a photosensitizing ligand. Given the 3D arrays of ultra‐small heavy metals, porous TZM serves as an efficient attenuator absorbing X‐ray energy and sensitizing hydroxyl radical generation for RT. Ta–Zr co‐doping narrowed the highest occupied molecular orbital‐lowest unoccupied molecular orbital (HOMO–LUMO) energy gap and exhibited close energy levels between the singlet and triplet photoexcited states, facilitating TZM transfer energy to the photosensitizer TCPP to sensitize singlet oxygen (^1^O_2_) generation for radiodynamic therapy (RDT). The sensitized RT–RDT effects of TZM elicit a robust antitumor immune response by inducing immunogenic cell death, promoting dendritic cell maturation, and upregulating programmed cell death protein 1 (PD‐L1) expression via the cGAS–STING pathway. Furthermore, a combination of TZM, X‐ray, and anti‐PD‐L1 treatments amplify antitumor immunotherapy and efficiently arrest osteosarcoma growth and metastasis. These results indicate that TZM is a promising radiosensitizer for the synergistic RT and immunotherapy of metastatic osteosarcoma.

## Graphical abstract

Ta–Zr co‐doped nanoscale metal−organic framework (nMOF) as a versatile radiosensitizer can offer significantly enhanced RT and RDT effects to elicit a robust antitumor immune response by activating the cGAS–STING pathway. Given its favorable properties of near‐infrared fluorescent/CT/PA multimodal imaging and good biocompatibility, this novel nMOF‐based radiosensitizer has potential applications in the efficient and precise treatment of osteosarcoma.

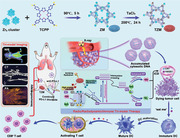

## 1 Introduction

Osteosarcoma is the most frequent primary bone tumor in children and adolescents worldwide, accounting for ≈56% of all bone sarcomas.^[^ ^1^ ^]^ The standard therapeutic regimens for localized osteosarcoma include surgical resection and systemic multiagent chemotherapy, which can achieve a 5‐year survival rate of ≈60%. Unfortunately, ≈10–15% of newly diagnosed osteosarcoma cases present with distant metastatic lesions, and the 5‐year overall survival rate (<20%) is unsatisfactory.^[^ ^1^, ^2^ ^]^ Currently, radiotherapy (RT) is an indispensable therapeutic strategy for patients with unresectable or metastatic osteosarcoma.^[^ ^3^ ^]^ RT utilizes ionizing radiation, such as X‐rays and *γ*‐rays, to kill cancer cells by directly inducing DNA damage, indirectly generating reactive oxygen species (ROS) to decompose biomolecules, or triggering an antitumor immune response by suddenly releasing tumor‐associated antigens and inducing immunogenic cell death (ICD).^[^ ^4^ ^]^ However, due to the domineering immunosuppressive microenvironment of cancer cells, low radiation doses often induce the radioresistance of tumor cells, whereas high radiation doses may cause adverse effects on immune cells and adjacent healthy tissues.^[^ ^5^ ^]^ Therefore, novel radiosensitizers that can significantly enhance the RT effect and synchronously elicit antitumor immunity are highly desirable.

Radiosensitizers can be classified into two main categories based on their structure: organic molecules (chemical compounds or biomacromolecules) and inorganic nanomaterials.^[^ ^6^ ^]^ In particular, metal‐based nanomaterials with high atomic (Z) elements, such as gold (Au),^[^ ^7^ ^]^ hafnium (Hf),^[^ ^8^ ^]^ and many lanthanide elements,^[^ ^9^ ^]^ have attracted considerable attention in recent decades for the development of radiosensitizers. Given their outstanding photoelectric and Compton effects, high‐Z metal elements can efficiently attenuate and absorb energy from X‐rays or *γ*‐rays to directly transfer to tumor cells or potentiate water radiolysis for ROS production.^[^ ^10^ ^]^ Among many nanoformulations, nanoscale metal−organic frameworks (nMOFs) have emerged as a unique class of inorganic–organic hybrid nanomaterials with several favorable advantages as radiosensitizers. First, with 3D arrays of ultrasmall heavy metal secondary building units, nMOFs afford superior radiosensitization over nonporous nanoparticles by more efficiently scattering secondary photons and electrons.^[^ ^11^ ^]^ Second, nMOFs are co‐assembled by organic ligands and high‐Z metal cluster nodes within a highly ordered structure, exhibiting large mass attenuation coefficients and strong dose enhancement effects.^[^ ^12^ ^]^ Third, the porosity of nMOFs is leveraged to encapsulate exceptionally high payloads of therapeutic and diagnostic cargoes, providing an opportunity for multimodal imaging‐guided synergistic tumor treatment.^[^ ^13^ ^]^ Last, the rational design and incorporation of some appropriate photosensitizing ligands in nMOFs can significantly enhance radiation damage to tumors via a unique radiotherapy–radiodynamic therapy (RT–RDT) process.^[^ ^14^ ^]^ Lin et al.^[^ ^11^, ^14^ ^]^ developed Hf‐based nMOFs for the RT–RDT of tumor cells. The high‐Z element Hf can absorb energy from X‐rays to potentiate water radiolysis (RT effect) and generate abundant singlet oxygen (^1^O_2_) (RDT effect) by transferring energy to photosensitizing linkers.^[^ ^15^ ^]^ The synergistic strategy not only significantly improves the RT effect but also enhances the photosensitization effect on deep‐tissue tumors excited using external soft X‐rays. In view of the multiple advantages mentioned earlier, developing efficient radiosensitizers from high‐atomic, metal‐based nMOF nanostructures is highly desirable.

**Scheme 1:**
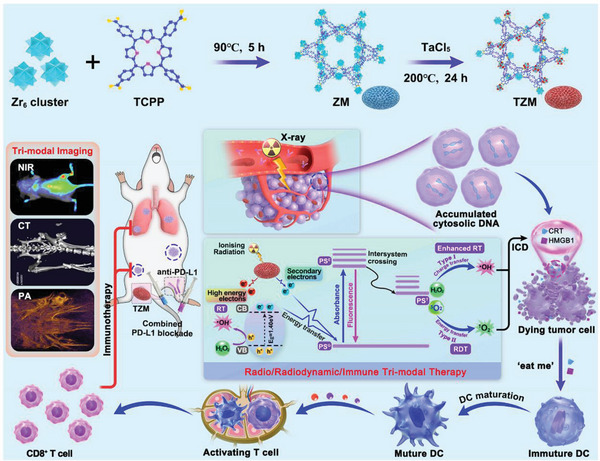
Schematic of TZM preparation and the proposed mechanism by which TZM sensitizes multi‐modal imaging‐guided RT/RDT/ immunotherapy for metastatic osteosarcoma. Upon X‐ray irradiation, Ta–Zr co‐doping facilitates X‐ray energy absorption by TXM to generate •OH from high‐Z Ta metals for RT. TZM also efficiently transfers energy to TCPP for •OH and ^1^O_2_ generation to enhance RT and RDT effects, respectively. The significantly sensitized RT and RDT effects kill the irradiated cancer cells and generate cytosolic DNA, which would be sensed by cGAS to activate the cGAS–STING pathway. This phenomenon increases the number of mature DCs and CD8^+^ T cells, finally evoking robust innate and adaptive immunity.

Tantalum (Ta) is a well‐known biomedical metal element widely used as a bone‐filling material,^[^ ^16^ ^]^ vascular stent,^[^ ^17^ ^]^ and contrast agent for computed tomography (CT) in clinics owing to its superb biocompatibility.^[^ ^18^ ^]^ Compared with the commercial radiosensitizer HfO_2_, Ta exhibits a higher X‐ray mass attenuation coefficient (4.30 cm^2^ kg^−1^ vs. 3.54 cm^2^ kg^−1^ at 100 keV) and higher atomic number (73 vs. 72), which confers its application prospect in CT imaging and RT–RDT.^[^ ^10b^ ^]^ Moreover, Ta can be used for photoacoustic (PA) imaging owing to its broad absorption properties and high photothermal conversion efficiency.^[^ ^19^ ^]^ However, to the best of our knowledge, no Ta‐based nMOFs have been reported as radiosensitizers for cancer treatment. Hence, this study aimed to design and develop a versatile nMOF (TZM) by incorporating high‐Z number elements Ta and Zr as metal nodes and TCPP as a photosensitizing ligand (**Scheme** **1**). Given the 3D arrays of ultrasmall heavy metals, porous TZM served as an efficient attenuator absorbing X‐ray energy and sensitizing hydroxyl radical (•OH) generation for RT. Ta–Zr co‐doping narrowed the highest occupied molecular orbital‐lowest unoccupied molecular orbital (HOMO–LUMO) energy gap and exhibited close energy levels between the singlet and triplet photoexcited states, facilitating energy transfer by TZM to the photosensitizer TCPP to sensitize ^1^O_2_ generation for RDT by promoting intersystem crossing (ISC) and nonradiative vibrational processes. By contrast, Zr‐MOF (ZM; without Ta incorporation) possessed a negligible RDT effect, and Ta‐TCPP (without Zr incorporation) could not even form nMOF nanostructures. Nanoscale TZM exhibited tumor‐preferential accumulation and near‐infrared (NIR) fluorescent/PA/CT tri‐modal imaging features that are helpful for imaging‐guided precise RT and real‐time monitoring of therapeutic response. With its enhanced RT–RDT effects and specific treatment of tumor cells, TZM elicited robust innate and adaptive antitumor immunity by inducing ICD, promoting the recruitment and activation of dendritic cells (DCs), and upregulating programmed cell death protein 1 (PD‐L1) expression via the cGAS–STING pathway. Consequently, a combination of TZM, X‐ray and anti‐PD‐L1 blockade amplified immunotherapy to efficiently arrest the growth and distant metastasis of osteosarcoma without any detectable toxicity. Therefore, TZM might serve as a brilliant radiosensitizer for the precise and efficient treatment of metastatic osteosarcoma.

## 2 Results and Discussion

### 2.1 Synthesis and Characterization of TZM

**Figure 1:**
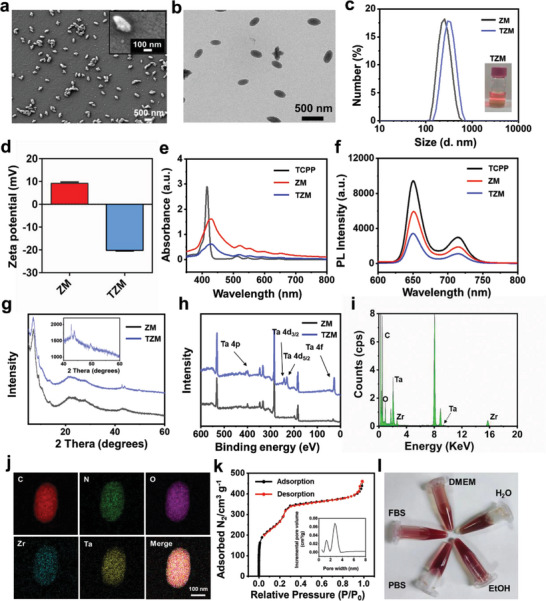
Morphological and compositional characterizations of TZM. a) SEM and b) TEM images of TZM. c) Hydrodynamic diameters and d) zeta potential of ZM and TZM measured using dynamic light scattering (n = 3). e) UV–vis absorption spectra and f) emission spectra of TCPP, ZM, and TZM (1 × 10^−5^ m) in H_2_O. g) XRD patterns and h) XPS spectra of ZM and TZM. i) Integrated EDX spectrum, j) TEM elemental mappings, and k) N_2_ adsorption–desorption isotherms of TZM. l) Representative digital photographs of TZM dispersed in various common solutions (H_2_O, EtOH, PBS, FBS, and DMEM). Data are presented as means ± standard deviations.

TZM was synthesized by incorporating Zr and Ta as metal nodes into nMOFs, in which TCPP was employed as a photosensitizing organic ligand by using the solvothermal method.^[^ ^20^ ^]^ Scanning electron microscopy (SEM) and transmission electron microscopy (TEM) images (**Figure** **1**a,b) showed that TZM exhibited a uniform monodispersed olive ball‐like shape ≈120 nm in width and 300 nm in length. Compared with ZM, TZM showed no distinctive changes in morphology, but its hydrodynamic size increased from 260.3 to 360.4 nm and zeta potential changed from 9.2 to −20.2 mV (Figure 1c,d; Figure S1a, Supporting Information). This result suggested that the incorporation of Ta can increase the particle size and induce a negative charge, which is consistent with a previous report that Ta nanoparticles have a negative charge.^[^ ^10b^ ^]^ Nevertheless, in the absence of Zr, we failed to obtain Ta‐MOF under the same solvothermal method (Figure S2, Supporting Information). These results indicate that Zr is a key metal node in the formation of nMOF nanostructures. The UV–vis absorption spectra of TCPP, ZM, and TZM showed a major Soret peak and four Q‐band characteristic absorption peaks (Figure 1e). However, TZM exhibited weaker photoluminescence than ZM and TCPP (Figure 1f). These results indicated that their photon absorption and electron–hole separation abilities upon light irradiation can facilitate metal–ligand charge transfer.^[^ ^21^ ^]^ The X‐ray diffraction (XRD) patterns (Figure 1g) showed that TZM possessed the same crystal structure as ZM at low angles of 5°–20° and displayed a new powder pattern of Ta at high angles of 40°–60°, indicating that amorphous TZM comprised crystalline ZM and Ta.^[^ ^22^ ^]^ The X‐ray photoelectron spectroscopy (XPS) spectra of TZM revealed the chemical valence values of metallic Ta at 28 (Ta4f7/2) and 26 eV (Ta4f5/2) (Figure 1h).^[^ ^19^, ^23^ ^]^ The energy dispersive spectroscopy patterns also confirmed that TZM comprised the expected essential metals Ta and Zr (Figure 1i). Furthermore, the HR‐TEM elemental mapping images (Figure 1j; Figure S1b, Supporting Information) exhibited that C, N, O, Zr, and Ta were uniformly distributed in the frameworks. Finally, the FT‐IR spectra (Figure S3, Supporting Information) showed a new peak at 673.52 cm^−1^, which can be attributed to the Ta—O bond.^[^ ^24^ ^]^ These results verified that Ta was successfully co‐incorporated with Zr into TCPP‐based nMOFs.

The Zr and Ta contents in TZM were determined using inductively coupled plasma mass spectroscopy (ICP‐MS) (Table S1, Supporting Information) to investigate the integration abilities of Zr and Ta. When the initial concentration of TaCl_5_ was increased from 2 to 10 mg mL^−1^, the Ta concentration in the framework increased from 139.3 ± 43.9 to 1171.2 ± 105.3 ng mL^−1^. However, it increased slightly to 1201.6 ± 72.0 ng mL^−1^ even when the concentration of TaCl_5_ reached 20 mg mL^−1^. Therefore, we selected the TaCl_5_ concentration of 10 mg mL^−1^ to prepare TZM unless otherwise mentioned. In the next TZM, the molar ratio of Ta to Zr was 4:1 as calculated using ICP‐MS. Compared with that of ZM, the specific surface area of TZM increased from 783.0 to 1061.3 m^2^ g^−1^ as determined by N_2_ adsorption–desorption isotherms, whereas no significant change in the average pore diameter was observed (TZM 2.40 nm vs. ZM 2.58 nm) (Figure 1k; Figure S4, Supporting Information). The results were consistent with the changes in particle size, repeatedly verifying that Ta and Zr were successfully co‐doped into the frameworks. TZM was well dispersed in various physiological solutions without visible precipitates, including H_2_O, phosphate buffer saline (PBS), fetal bovine serum (FBS), and Dulbecco's Modified Eagle's Medium (DMEM) supplemented with 10% FBS (Figure 1l). Furthermore, the prepared TZM showed no obvious changes in hydrodynamic particle size after 5 days of incubation in H_2_O, indicating its good stability and feasibility for further biological evaluation (Figure S5, Supporting Information). The enhanced water stability in metal‐doped MOFs may be ascribed to the fact that the doped metal ions (Ta^5+^) perturb the ligand surface, hindering the formation of water clusters in the pores.^[^ ^25^ ^]^

### 2.2 TZM‐Induced ROS Generation under X‐Ray Irradiation

**Figure 2:**
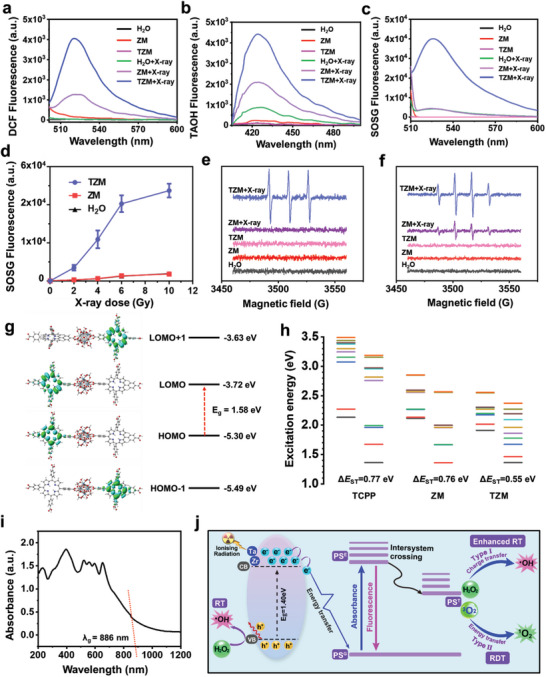
Radio‐radiodynamic efficacies of TZM in vitro. a) Fluorescence spectra of a) DCF, b) TAOH, and c) SOSG for detecting total ROS, •OH, and ^1^O_2_ generation in different groups with or without X‐ray irradiation (6 Gy). d) Fluorescence intensity of SOSG for detecting ^1^O_2_ after gradually increasing X‐ray radiation dose. ESR spectra for e) ^1^O_2_ and f) •OH detection under X‐ray irradiation in the presence of spin trap TEMP and DMPO, respectively. g) DFT calculation of frontier molecular orbitals and corresponding energy levels of TZM. h) Calculated excitation energy distributions of singlet (Sn) and triplet (Tn) excited states for TCPP, ZM, and TZM. i) UV–vis diffuse reflectance spectrum of TZM. j) Schematic of the mechanism of the RD–RDT effects of TZM.

Considering the existence of two high‐Z metal elements (Ta and Zr) and a photosensitizing linker (TCPP), we hypothesized that TZM can serve as an effective RT–RDT inducer. Among all groups, the TZM + X‐ray group exhibited the strongest fluorescence intensities of 2′,7′‐dichlorodihydrofluorescein (H_2_DCF) and disodium 2‐hydroxy terephthalate (TAOH) for ROS detection (**Figure** **2**a,b). This result indicates that total ROS and •OH generation were the highest in the TZM + X‐ray group, respectively. Moreover, only the TZM + X‐ray group exhibited apparent fluorescence signal peaks of singlet oxygen sensor green (SOSG) for ^1^O_2_ detection (Figure 2c). The generation of ^1^O_2_ increased with increasing X‐ray dose in the TZM group but not in the H_2_O or ZM group (Figure 2d). Meanwhile, ^1^O_2_ generation obviously increased as the Ta content in the TZM system was increased up to 1171.2 ± 105.3 ng mL^−1^ (Figure S6, Supporting Information). ^1^O_2_ generation by TZM under X‐ray irradiation was further evaluated using electron spin resonance (ESR). Consistent with the aforementioned results, the typical 1:1:1 peak for ^1^O_2_ was detected in the TZM X‐ray group but not in the H_2_O and ZM groups after X‐ray irradiation (Figure 2e). The typical 1:2:2:1 peak for •OH was detected in the ZM and TZM X‐ray groups; however, the peak intensity in the TZM group was significantly stronger than that in the ZM group (Figure 2f). These findings revealed that TZM can generate high amounts of ^1^O_2_ and •OH owing to the overwhelming contribution of Ta. Regarding the pivotal role of Zr in the formation of nMOF nanostructures, co‐doping Ta and Zr into an nMOF were crucial to potentiate RT–RDT effects. Density functional theory (DFT) and time‐dependent (TD‐DFT) calculations were performed using the Gaussian 09 package to study the molecular properties of TZM and ZM. As presented in Figure 2g, the HOMO–LUMO bandgap energy (*E*_g_) values of TZM and ZM were calculated to be 1.58 and 2.66 eV, respectively (Figure S7, Supporting Information). A semiconductor with a narrow bandgap usually exhibits long‐wavelength absorption and a high quantum yield of ROS.^[^ ^15^, ^26^ ^]^ In addition, TZM in the singlet (Sn) and triplet (Tn) excited states is beneficial for the ISC of TCPP. The energy gap (Δ*E*_ST_) between S1 and T1 was reduced from TCPP (0.77 eV) to TZM (0.55 eV), as shown in Figure 2h. Compared with TCPP and ZM, TZM exhibited closer energy levels between the singlet and triplet photoexcited states (Figure 2h). The *E*_g_ value of TZM was calculated to be 1.40 eV (Figure 2i) through UV photoelectron spectroscopy (absorption threshold *λ*_g_ = 886 nm) as previously described (Figure S8, Supporting Information).^[^ ^27^ ^]^ This value is significantly lower than those of various well‐known inorganic radiosensitizers (e.g., ZrO_2_, 5.00 eV; WO_3_, 2.70 eV; and YFeO_3_, 2.70 eV). Hence, TZM was calculated with a narrow HOMO–LUMO energy gap and a small Δ*E*_ST_. As illustrated in Figure 2j, TZM can generate abundant ROS in two ways under X‐ray irradiation. On the one hand, Ta as a high‐Z element can efficiently absorb energy from X‐rays to potentiate water radiolysis and generate tumoricidal •OH for the RT effect. On the other hand, under X‐ray excitation, the generated electrons of Ta^5+^ ions can excite nearby Zr^4+^ ions to transfer their energy to porphyrinic photosensitizers (TCPP), promoting the generation of abundant triplet photoexcited states through ISC and the generation of abundant ^1^O_2_ (for RDT) and •OH (for enhanced RT) through nonradiative vibrational processes. The ground state (PS^G^) of TCPP was initiated to an excited singlet state (PS^E^) with energy transfer, followed by ISC to an excited triplet state (PS^T^). Finally, PS^T^ returned to PS^G^, accompanied by the generation of abundant ^1^O_2_ and •OH through type I (electron transfer) and type II (energy transfer) reactions, respectively.^[^ ^12^ ^]^

### 2.3 RT–RDT–ICD Effects of TZM In Vitro

**Figure 3:**
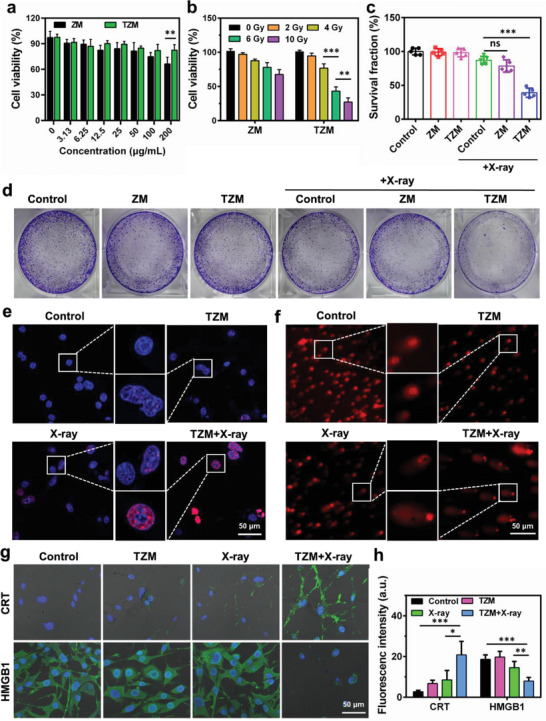
In vitro therapeutic efficacy of TZM. a) Cytotoxicity studies through CCK‐8 assay of K7M2 cells incubated with various concentrations of ZM and TZM (n = 3). b) Relative viability of K7M2 cells after treatment with ZM and TZM under different X‐ray irradiation doses (n = 3). c) Survival fraction and d) colony formation of K7M2 cells incubated with PBS, ZM, and TZM with or without X‐ray irradiation (6 Gy) (n = 5). e) DNA double‐strand breaks in K7M2 cells incubated with PBS or TZM with or without X‐ray irradiation (6 Gy). Nuclei were stained with Hoechst 33 342 (blue) and *𝛾*‐H2AX (red). f) Representative comet assay images of cells induced by TZM under X‐ray irradiation (6 Gy). g) Fluorescence images of CRT‐ or HMGB1‐stained K7M2 cells after incubation with PBS or TZM with or without X‐ray irradiation (6 Gy). h) Semi‐quantitative analysis of fluorescence images of CRT and HMGB1 (n = 10). Data are presented as mean ± standard deviation. Student's *t*‐test or one‐way ANOVA with Tukey's post hoc test was performed. “ns” represents no significance, *p < 0.05, **p < 0.01, ***p < 0.001.

Cell counting kit‐8 assay results revealed that TZM, even at high concentrations (up to 200 µg mL^−1^), did not cause obvious cytotoxicity against cancer cells (**Figure** **3**a). In subsequent experiments, 100 µg mL^−1^ was selected as the optimal concentration for further tests, unless otherwise mentioned. By contrast, ZM exhibited over 20% cytotoxicity against K7M2 cells at a high concentration of 200 µg mL^−1^. This result can be ascribed to the fact that the more biocompatible Ta partially replaced Zr and co‐doped in MOFs to reduce toxicity. The cellular uptake of TZM was evaluated in K7M2 osteosarcoma cells through confocal microscopy. The internalization of TZM by K7M2 cells increased in a time‐dependent manner and peaked at 12 h until 36 h after incubation (Figure S9, Supporting Information).

Encouraged by the excellent ROS generation efficacy of TZM under X‐ray irradiation, we evaluated its in vitro anticancer efficacy. As shown in Figure 3b, the relative viability of the TZM‐treated cells decreased sharply with increasing X‐ray irradiation dose. However, no significant difference in relative viability was found between the cells in the ZM + X‐ray and X‐ray groups, indicating that the co‐incorporation of Ta–Zr in the nMOF structure significantly enhanced the RT effect. We also performed a supplementary experiment to confirm whether the radiosensitization effect of TZM is reproducible in human osteosarcoma U2OS cells (Figure S10, Supporting Information). As expected, our result revealed that TZM also exhibited an excellent radiosensitizing effect against U2OS cells. We then evaluated cell death through flow cytometry following Annexin V/PI staining (Figure S11, Supporting Information). The percentage of apoptotic cells was higher in the TZM + X‐ray group (56.6%) than in the TZM (1.4%) and X‐ray (17.0%) groups. Consistent with the apoptosis results, TUNEL staining also exhibited the strongest fluorescence signal in the TZM + X‐ray group (Figure S12, Supporting Information). Colony formation assays confirmed that the cells in the TZM + X‐ray group exhibited the lowest fraction (Figure 3c,d). To further verify the radiosensitization effect of TZM, we examined DNA double‐strand breaks by using *γ*‐H2AX immunofluorescence staining (Figure 3e; Figure S13, Supporting Information), a commonly used gold standard marker for DNA damage detection. As anticipated, the most apparent *γ*‐H2AX foci were observed in the nuclei of the cells in the TZM + X‐ray group, indicating maximum DNA damage. Similarly, comet assay results also verified that the amount of DNA fragments was larger in the TZM + X‐ray group than in the other groups (Figure 3f; Figure S14, Supporting Information). The percentage of tail DNA content in the TZM + X‐ray group was calculated to be 49.0% ± 8.8%, which was three times higher than that in the X‐ray group (16.5% ± 3.3%). Furthermore, the generation of ROS, SOSG, and malondialdehyde in K7M2 cells was determined by confocal microscopy after the indicated treatments (Figure S15, Supporting Information). Notably, the most intense green fluorescence appeared in the cells from the TZM + X‐ray group, which might be explained by the excellent ability of TZM to generate cytotoxic ROS.

The expression of calreticulin (CRT) and the release of high‐mobility group box‐1 (HMGB1) as critical ICD markers^[^ ^28^ ^]^ were detected to evaluate whether the sequentially enhanced RT–RDT effects of TZM can trigger a positive antitumor immune response (Figure 3g,h). Among the cells from the various groups, the cells from the TZM + X‐ray group exhibited the strongest fluorescence intensity for CRT (green) and the weakest fluorescence intensity for HMGB1 (red), suggesting the successful exposure of CRT on the plasma membrane and the exhaustive release of HMGB1 from cells. Additionally, the release of HMGB1 into the supernatants was detected using an ELISA kit (Figure S16, Supporting Information). HMGB1 was released from the nucleus into the extracellular environment after TZM + X‐ray treatment. Both ICD markers can act as “eat‐me” dangerous signals to stimulate immature DCs and macrophages to engulf dying tumor cells, thus enhancing antigen presentation and activating T cells.^[^ ^29^ ^]^ Collectively, these results verified that TZM significantly sensitized RT and RDT effects to induce the robust ICD of K7M2 osteosarcoma cells.

### 2.4 NIR Fluorescent/CT/PA Imaging Properties of TZM

**Figure 4:**
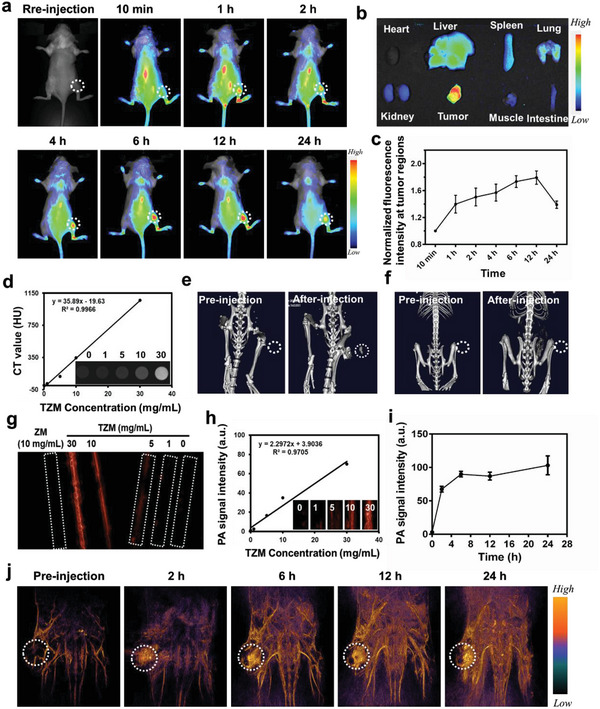
Tri‐modal imaging properties of TZM in vivo. a) In vivo NIR fluorescence imaging of tumor‐bearing mice taken at different time points post TZM intravenous injection. b) Ex vivo fluorescence images of vital organs (i.e., heart, liver, spleen, lung, kidney, muscle, and intestine) and tumor tissues collected from mice 24 h after TZM intravenous injection. c) Semi‐quantitative analysis of fluorescence intensities at the tumor site of tumor‐bearing mice (n = 3). d) In vitro CT images and curve (Hounsfield unit vs. mass concentration) of TZM at various gradient concentrations (0–30 mg mL^−1^). In vivo CT imaging of the tumor region 12 h after intravenous injection of e) TZM and f) ZM (10 mg mL^−1^). g) PA signals of ZM and TZM in aqueous solution under 808 nm laser excitation. h) Linear relationship between TZM concentration and PA intensity in aqueous solution (0–30 mg mL^−1^). i,j) Semi‐quantitative analysis (n = 3) and PA signal in the tumor were detected after TZM intravenous injection (10 mg mL^−1^). Data are presented as mean ± standard deviation.

Precisely distinguishing tumors from their boundaries is important to maximize cancer cell killing and minimize side effects. Molecular imaging techniques (e.g., fluorescence imaging, magnetic resonance imaging, and CT) offer great convenience for precise imaging‐guided RT.^[^ ^30^ ^]^ In the present study, we characterized the cancer‐targeting ability and bio‐imaging potential of TZM. Considering that TCPP is a photosensitizer with high NIR light absorption and emission, we evaluated the tumor retention and fluorescent imaging properties of TZM in a tumor‐bearing BABL/c model using a NIR small‐animal imaging system.^[^ ^31^ ^]^ Red fluorescence signals appeared at the tumor site 1 h after intravenous injection and gradually peaked at 12 h (**Figure** **4**a,c; Figure S17, Supporting Information). Ex vivo imaging of the harvested tumor tissue and vital organs (i.e., heart, liver, spleen, lung, kidney, muscle, and intestine) revealed the strongest fluorescence intensity in the tumor (Figure 4b; Figure S18, Supporting Information). The biodistribution and pharmacokinetic properties of TZM in the tumor‐bearing mice were determined by measuring Ta concentration through ICP‐MS. As presented in Figure S19 (Supporting Information), the TZM concentration in the blood remained 5.7% ± 3.5% ID g^−1^ even at 48 h after intravenous injection, indicating a long blood circulation time. Meanwhile, the accumulation of Ta at the tumor site increased and peaked 12 h after injection, which is consistent with the results of NIR fluorescent imaging. Probably because of tumor vascular enhanced permeability and retention and nanoparticle shape effect, the nanoscale TZM has an average size of ≈120 nm in width (20–200 nm) and a uniform monodispersed olive ball‐like shape similar to nanoshuttles, which are beneficial for tumor‐preferential accumulation.^[^ ^32^ ^]^ Considering that Ta can efficiently attenuate X‐ray energy and serve as a candidate contrast agent in CT,^[^ ^33^ ^]^ we explored the CT imaging properties of TZM. A positive linear relationship was found between TZM concentration and CT signal value (R^2^ = 0.9966); in specific, CT signal intensity increased with increasing TZM concentration (Figure 4d). As expected, no CT signals were detected in the tumor site before TZM administration, whereas strong CT signals from the tumor site were observed 12 h after intravenous injection (Figure 4e), indicating the feasibility of TZM for tumor CT imaging. By contrast, in vitro, and in vivo studies showed that ZM did not exhibit distinct CT signals (Figure 4f; Figure S20, Supporting Information). Furthermore, TZM exerted obvious PA intensities under 808 nm laser excitation, whereas ZM did not (Figure 4g). The distinct differences in CT and PA performances between TZM and ZM can be mainly ascribed to the efficient X‐ray attenuation and NIR absorption properties of Ta. The PA intensities of TZM under 808 nm laser emission increased linearly with increasing concentration (Figure 4h). Based on the good response of PA signals in solution, we evaluated the PA imaging performance of TZM in tumor‐bearing mice. As presented in Figure 4i,j and Video S1 (Supporting Information), the PA signal intensities of TZM at the tumor site were clearly observed 2 h after injection, gradually increased over time, and then peaked at 12 h, suggesting a favorable potential of TZM for PA imaging. Overall, these results demonstrate that TZM is versatile for tumor‐preferential accumulation and a potential tri‐modal imaging contrast agent.

### 2.5 RT/RDT/Immunotherapy Effects of TZM In Vivo

Inspired by the favorable RT/RDT effects in vitro and tumor‐preferential accumulation in vivo of TZM, we initially evaluated the antitumor efficiency of TZM in vivo in a unilateral tumor model. When the tumor volume reached ≈100 mm^3^, the mice were randomly divided into four groups: PBS, TZM, X‐ray, and TZM + X‐ray. TZM was intravenously injected into the mice thrice at an interval of 1 day, and only the tumor region was subjected to X‐ray irradiation (6 Gy × 3) 12 h after injection (Figure S21, Supporting Information). The mice from the various groups showed no apparent changes in body weight during the 14‐day treatment period (Figure S22a, Supporting Information), suggesting the negligible systemic toxicity of the various treatments. No apparent tumor growth inhibition was observed in the mice from the TZM group (Figure S22b,c, Supporting Information). By contrast, tumor growth was efficiently inhibited and tumor burden was the lowest in the mice from the TZM + X‐ray group. This result can be ascribed to the direct effects of RD–RDT on tumors mediated by TZM. A similar trend in tumor weight was observed (Figure S22d, Supporting Information). Hematoxylin and eosin (H&E) staining results confirmed that the tumors in the mice from the TZM + X‐ray group were seriously destroyed, as evidenced by the large‐area tumor necrosis and acellular regions (Figure S23, Supporting Information). Moreover, immunohistochemical staining of tumor slices supported that the percentage of Ki‐67‐positive cells was the lowest and the number of TUNEL‐positive cells was the highest in the mice from the TZM + X‐ray group (Figure S23, Supporting Information).

**Figure 5:**
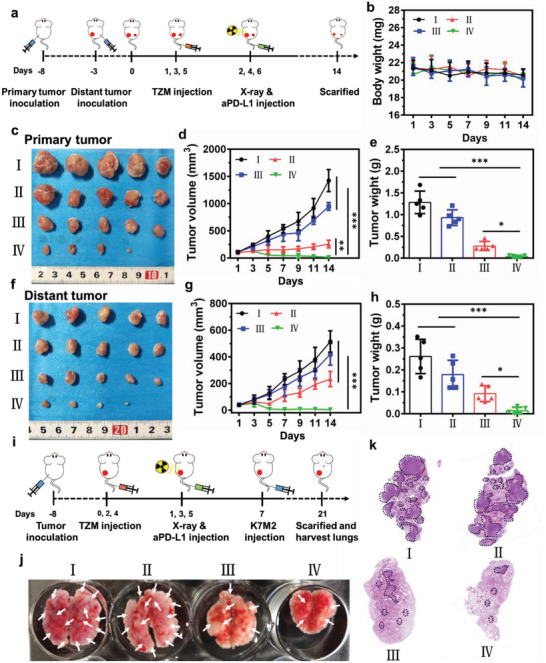
Therapeutic effects of TZM‐mediated RD–RDT–immunotherapy on distant tumor and lung metastasis. a) Schematic of the experimental process for bilateral K7M2 tumor‐bearing mice. b) Body weights of the mice after receiving the indicated treatments (n = 5). c,f) Isolated tumor digital photographs, d,g) tumor volume curves, and e,h) primary and distant tumor weights in various groups after receiving indicated treatments, respectively (n = 5). i) Schematic of the experimental process for lung metastasis model. j) Representative digital photographs and k) H&E staining of lung tissues in various groups after receiving indicated treatments. For each group: I) PBS; II) anti‐PD‐L1 + X‐ray; III) TZM + X‐ray; IV) TZM + X‐ray + anti‐PD‐L1. Data are presented as mean ± standard deviation. Statistical analysis was performed using one‐way ANOVA with Tukey's post hoc test, *p < 0.05, **p < 0.01, ***p < 0.001.

Our previous in vitro results revealed that the combination of TZM and X‐ray irradiation induces ICD. Therefore, we hypothesized that the combination of TZM, X‐ray, and anti‐PD‐L1 treatments can synergistically inhibit distant tumor growth and metastasis. To verify this hypothesis, we evaluated the antitumor effect of combination therapy in a bilateral tumor‐bearing mouse model (**Figure** **5**a). No obvious changes in body mass were observed in the mice from the various groups, suggesting good tolerance and unobvious side effects of this treatment (Figure 5b). Compared with PBS, anti‐PD‐L1 + X‐ray treatment only slightly suppressed tumor growth, whereas TZM + X‐ray treatment significantly inhibited tumor growth (*p* < 0.001). Among the treatments, TZM + X‐ray + anti‐PD‐L1 afforded the most significant tumor suppression (Figure 5c–e). The growth of distant tumors was similar to that of primary tumors (Figure 5f–h). H&E, Ki‐67, and TUNEL staining confirmed that primary and distant tumor cells were severely apoptotic and destroyed with apparent morphological changes in the TZM + X‐ray and anti‐PD‐L1 groups (Figures S24, S25, Supporting Information). This phenomenon might be explained by the neutralization of immune escape routes by PD‐L1 blockade and boosting of systemic antitumor immunity.

Considering the aggressive nature of osteosarcoma tumor cells that frequently metastasize to the lungs,^[^ ^34^ ^]^ we established a pulmonary metastasis model to intuitively evaluate the joint antitumor metastasis efficacy of TZM‐mediated RT–RDT and immunotherapy. As illustrated in Figure 5i, K7M2 cells were intravenously injected into unilateral tumor‐bearing mice 7 days after receiving the indicated treatments. Lung tissues were excised from the mice on day 21, and lung metastasis was examined through metastatic nodule counting and H&E staining. As shown in Figure 5j,k and Figure S26 (Supporting Information), the number of nodules was fewer in the lungs of the mice from the TZM + X‐ray group than in those of the mice from the PBS and anti‐PD‐L1 + X‐ray groups. As expected, the fewest nodules were found in the lungs of the mice from the TZM + X‐ray + anti‐PD‐L1 group. These results again verify that TZM + X‐ray treatment can induce a robust antitumor immune response and that this effect can be amplified by adding anti‐PD‐L1 treatment.

### 2.6 Mechanisms Underlying the Antitumor Immune Response Activated by the RT–RDT Effects of TZM

**Figure 6:**
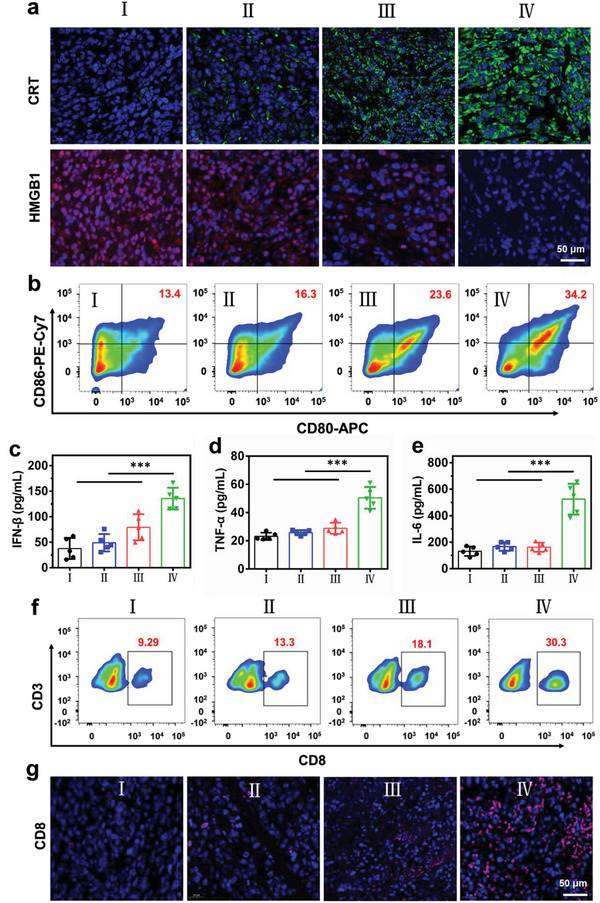
TZM‐mediated RT–RDT stimulates antitumor immunity in mice. a) CRT and HMGB expression in tumor slices excised 3 days from the mice after receiving the indicated treatments. b) Flow cytometry of mature DC cells (CD80^+^ and CD86^+^) in the lymph nodes from tumor‐bearing mice isolated on day 3 after receiving the indicated treatments. c) Serum levels of IFN‐*β*, d) TNF‐*α*, and e) IL‐6 3 days after receiving the indicated treatments (n = 5). f) Flow cytometry and g) immunofluorescence analysis of tumor‐infiltrating CD8^+^ (CD3^+^ and CD8^+^) T cells 5 days after receiving the indicated treatments. For each group: I) PBS; II) anti‐PD‐L1 + X‐ray; III) TZM + X‐ray; IV) TZM + X‐ray + anti‐PD‐L1. Data are presented as mean ± standard deviation. Statistical analysis was performed using one‐way ANOVA with Tukey's post hoc test, ***p < 0.001.

To elucidate the mechanisms underlying the in vivo antitumor immune response activated by the RT–RDT effects of TZM, we explored the expression of ICD indicators, maturation of DCs, and a number of tumor‐infiltrating cytotoxic T cells in tumor tissues and lymph nodes. As shown in **Figure** **6**a, the tumor slices from the mice in the TZM + X‐ray group showed the highest CRT exposure (green fluorescence). The combination treatment also caused the release of a large amount of HMGB1 from the nucleus into the extracellular matrix, indicating that the combination treatment can also efficiently trigger ICD in vivo. The percentage of mature DCs (CD80^+^ CD86^+^) in the lymph nodes was determined (Figure 6b; Figure S27, Supporting Information). Correspondingly, the frequency of CD80^+^ and CD86^+^ DCs was moderately increased in the TZM + X‐ray group; in specific, it was 1.8‐fold higher in the TZM + X‐ray group than in the PBS group. Meanwhile, the population of mature DCs was the highest in the TZM + X‐ray + anti‐PD‐L1 group; it was 2.4‐fold higher in the TZM + X‐ray + anti‐PD‐L1 group than in the PBS group, suggesting the strong recruitment and activation of DCs.

We determined the levels of immune‐associated cytokines, such as interferon (IFN)‐*β*, tumor necrosis factor (TNF)‐*α*, and interleukin (IL)‐6, in tumors after the specified treatments. All three cytokines were significantly elevated after TZM + X‐ray + anti‐PD‐L1 treatment (Figure 6c–e). The population of activated CTLs in distant tumors was also explored. Flow cytometry results (Figure 6f; Figure S29, Supporting Information) showed that TZM + X‐ray treatment recruited a 1.9‐fold higher percentage of tumor‐infiltrating CD8^+^ T lymphocytes (CD3^+^ and CD8^+^) than PBS treatment, whereas TZM + X‐ray + anti‐PD‐L1 treatment recruited a 3.2‐fold higher percentage of CD8^+^ T lymphocytes than PBS treatment. The immunofluorescence staining results further demonstrated that a large number of CD8^+^ T cells infiltrated all regions of the tumor tissue in the TZM + X‐ray + anti‐PD‐L1 group (Figure 6g). The above results uncover a preliminary antitumor immune mechanism in which the outstanding RT–RDT effects of TZM can elicit a robust systemic immune response and the addition of anti‐PD‐L1 treatment can further amplify antitumor immunity.

**Figure 7:**
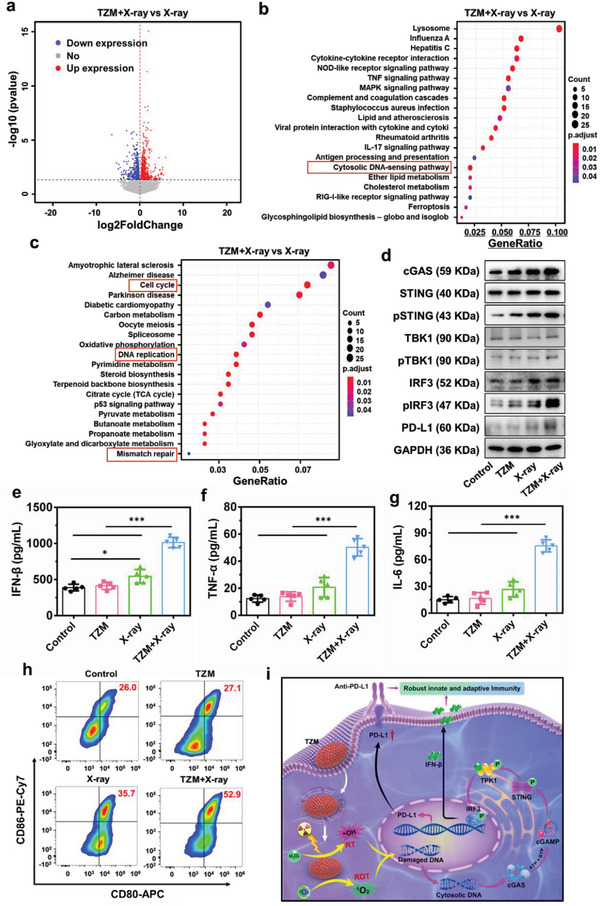
TZM‐mediated RT–RDT effects may trigger antitumor immunity by activating the cGAS/STING signaling pathway and upregulating PD‐L1 expression. a) Volcano plot showing the DEGs in the X‐ray and TZM + X‐ray groups. GO enrichment analysis of the gene functions of b) the upregulated and c) downregulated DEGs. d) Western blot analysis of the expression of cGAS–STING‐TBK1‐IRF3 signaling‐related genes in K7M2 cells incubated with PBS and TZM with or without X‐ray irradiation (6 Gy). e) ELISA analysis of the release of IFN‐*β* from tumor cells in various groups (n = 5). Cytokine secretion of TNF‐*α* (f) and IL‐6 (g) in the supernatant of bone marrow‐derived cells (BMDCs) (n = 5). h) Percentage of mature DCs after incubation with supernatant harvested from PBS and TZM with or without X‐ray irradiation (6 Gy). i) Schematic of the cGAS/STING signaling pathway activated by TZM with X‐ray irradiation. Data are presented as mean ± standard deviation. Statistical analysis was performed using one‐way ANOVA with Tukey's post hoc test, *p < 0.05 and ***p < 0.001.

We intended to elucidate the in‐depth mechanisms underlying the antitumor immune response activated by the RT–RDT effects of TZM through transcriptome sequencing. Quantile normalization was applied to the fragment per kilobase per million mapped reads (Student's *t*‐test at *p* = 0.05), and then‐candidate genes displaying a differential expression of at least two‐fold change compared with that in the control group were selected. As shown in **Figure** **7**a, 1205 differentially expressed genes (DEGs) (632 upregulated and 573 downregulated) with fold change > 2 and *p* < 0.05 were identified between the X‐ray and TZM + X‐ray groups. Kyoto Encyclopedia of Genes and Genomes pathway enrichment analysis revealed that these DEGs were predominantly involved in the cell cycle, DNA damage repair, and immune‐related signaling pathways (Figure 7b,c). Gene ontology (GO) enrichment analysis also confirmed that the DEGs participated in multiple biological processes, cellular components, and molecular functions (Figure S31, Supporting Information). These results confirmed that TZM‐mediated RT–RDT effects can effectively lead to DNA damage, and the various DEGs were associated with the cytosolic DNA‐sensing pathway. Damaged dsDNA can be released from the nucleus to the cytoplasm, subsequently stimulating antitumor immune responses by activating the cGAS/STING signaling pathway.^[^ ^35^ ^]^ Thus, we hypothesized that the RT–RDT effects of TZM trigger an antitumor immune response by activating the cGAS/STING signaling pathway. To verify this hypothesis, the protein expression levels of cGAS/STING pathway‐related genes (cGAS, STING, TBK1, and IRF3) were measured in the cells after the indicated treatments (Figure 7d; Figures S32, S33, Supporting Information). Western blot results demonstrated that the expression levels of cGAS, p‐STING, p‐TBK1, and p‐IRF3 were remarkably higher in the TZM + X‐ray group than in the three other groups, indicating the increased phosphorylation of these indicators and activation of the cGAS/STING pathway. According to previous reports,^[^ ^36^ ^]^ the cGAS/STING pathway plays a crucial role in RT‐induced anti‐tumor immune responses by contributing to PD‐L1 upregulation. Therefore, we examined whether TZM‐mediated RT–RDT could alter the expression profile of PD‐L1 in tumor cells. As shown in Figure 7d, PD‐L1 expression was only slightly upregulated in the control and TZM non‐irradiated groups but was upregulated twofold in the TZM + X‐ray group. The PD‐L1 upregulation might also explain why the best antitumor immune response against metastatic osteosarcoma was achieved under TZM + X‐ray + anti‐PD‐L1 treatment.

Initiation of the STING‐dependent response can enhance the production and release of multiple pro‐inflammatory cytokines.^[^ ^37^ ^]^ In the present study, IFN‐*β* secretion was analyzed in K7M2 cells subjected to TZM + X‐ray treatment. Results showed that TZM + X‐ray treatment induced IFN‐*β* secretion (Figure 7e) and significantly increased TNF‐*α* and IL‐6 levels (Figure 7f,g). In view of the fact that mature DCs can amplify the cancer immunity cycle by producing multiple pro‐inflammatory cytokines,^[^ ^38^ ^]^ we evaluated the maturation of DCs (Figure 7h; Figure S34, Supporting Information). As anticipated, the percentage of mature DCs (CD80^+^ and CD86^+^) in the TZM + X‐ray group was notably elevated and quantified to be 52.3% ± 2.3%, which was 1.9‐ and 1.5‐fold higher than those in the TZM (27.3% ± 1.2%) and X‐ray (35.3% ± 1.7%) groups, respectively. Collectively, these results suggest that the RT–RDT effects of TZM induce ICD‐mediated damage‐associated molecular pattern release and cGAS–STING‐mediated cytokine secretion to stimulate DC maturation and PD‐L1 upregulation, which subsequently evoke robust antitumor immune responses (Figure 7i).

### 2.7 Biosafety Assessment of TZM

Good biocompatibility is a prerequisite of any chemical drug or advanced nanocomposite for practical clinical applications. We systematically administered TZM (20 mg kg^−1^) to healthy mice and evaluated its biosafety. The mice from the various groups showed no apparent difference in body mass during the 14‐day treatment (Figure S35, Supporting Information). All blood routine and biochemical parameters of the mice on days 1, 7, and 14 after administration were detected and found to be within the normal reference limits (Figures S36,S37, Supporting Information). Moreover, H&E staining of the major organs from the PBS‐ or TZM‐treated mice revealed no evident pathological abnormalities (Figure S38, Supporting Information). Hemocompatibility assay results also indicated that the hemolysis rate was 3.2% after treatment with TZM, even at a high concentration of 200 µg mL^−1^. By contrast, the hemolysis rate after treatment with 100 µg mL^−1^ ZM was 7.0% (Figure S39, Supporting Information). This result may be explained by the excellent biocompatibility of Ta.^[^ ^19^ ^]^ The aforementioned results suggest that our synthesized TZM possesses favorable biocompatibility, providing promising potential for translational applications. The better biocompatibility and more efficient RT–RDT effects of TZM than ZM also explicitly highlight the importance of our proposed Ta–Zr co‐doping strategy.

Currently, RT and immunotherapy are the two main clinical therapeutic strategies for patients with unresectable or metastatic osteosarcoma. However, due to the domineering immunosuppressive microenvironment, low radiation doses often induce radioresistance, whereas high radiation doses cause undesirable adverse effects on immune cells. Therefore, effective and precise radiosensitizers need to be developed to broaden the therapeutic window and achieve long‐term outcomes for patients. In this study, we rationally designed and successfully fabricated a versatile radiosensitizer (TZM) with RT–RDT–immunotherapy synergistic effects and good biocompatibility for metastatic osteosarcoma treatment.

TZM was synthesized using the solvothermal method by incorporating high‐atomic‐number elements Zr and Ta as metal nodes into MOFs, in which photosensitizing TCPP was employed as the organic ligand. With 3D arrays of high‐Z elements Ta and Zr as metal nodes, porous TZM afforded superior radiosensitization by scattering secondary photons and electrons efficiently. Ta–Zr co‐doping facilitated not only X‐ray energy absorption by TZM to generate •OH from high‐Z Ta metals for RT but also energy transfer from TZM to TCPP via Zr, efficiently generating ^1^O_2_ and •OH to achieve RDT and enhanced RT effects, respectively. ZM exhibited negligible RDT effects, whereas Ta‐TCPP could not even form nMOF nanostructures, indicating the importance of Ta–Zr co‐doping.

Owing to the sequentially sensitized RT–RDT effects, TZM plus X‐ray treatment elicited a robust antitumor immune response by inducing ICD, promoting the recruitment and activation of DCs. With RT–RDT–immunotherapy effects, TZM efficiently suppressed the growth of primary osteosarcoma tumors and generated favorable abscopal effects to inhibit distant metastatic osteosarcoma in a bilateral tumor‐bearing mouse model and a pulmonary metastasis model. Antitumor immunotherapy was further enhanced by the combination of TZM, X‐ray, and anti‐PD‐L1 blockade treatments. The in‐depth mechanisms underlying the antitumor immunity response induced by TZM were investigated. Results revealed that the sensitized RT‐RDT effects of TZM caused the release of a large amount of damaged dsDNA from the nucleus to the cytoplasm. The damaged dsDNA subsequently stimulated antitumor immune response by activating the cGAS–STING pathway and inducing PD‐L1 upregulation. Owing to the imaging properties of TCPP and Ta, nanoscale TZM also exhibited NIR fluorescence/PA/CT tri‐modal imaging features. Thus, TZM can be developed as a cancer theranostic agent for imaging‐guided precise RT and real‐time monitoring of therapeutic response.

## 3 Conclusion 

In summary, we developed a Ta–Zr co‐doped nMOF (TZM) as a versatile radiosensitizer for the first time. Results of in vitro and in vivo experiments involving two animal models with distant metastatic osteosarcoma revealed that TZM can offer significantly enhanced RT and RDT effects and elicit a robust antitumor immune response. Given its favorable properties of NIR fluorescent/CT/PA multimodal imaging and good biocompatibility, this novel nMOF‐based radiosensitizer has potential applications in the efficient and precise treatment of osteosarcoma.

## Conflict of Interest

The authors declare no conflict of interest.

## Supporting information

Supporting information

Supplemental Video 1

## Data Availability

The data that support the findings of this study are available from the corresponding author upon reasonable request.
