# Supplementary material for: Tantalum–Zirconium Co‐Doped Metal–Organic Frameworks Sequentially Sensitize Radio–Radiodynamic–Immunotherapy for Metastatic Osteosarcoma
Source: Adv Sci (Weinh). 2023 Feb 5;10(10):2206779. doi: 10.1002/advs.202206779 (PMC10074130; doi:10.1002/advs.202206779)
Supplement: Supplementary file 1 — Supporting information [file ADVS-10-2206779-s001.pdf]

Supporting Information

Tantalum–Zirconium Co-doped Metal－Organic Frameworks Sequentially Sensitize Radio–Radiodynamic–Immunotherapy for Metastatic Osteosarcoma

Tao Li, Mingquan Gao, Zifei Wu, Junjun Yang, Banghui Mo, Songtao Yu, Xiaoyuan Gong, Jing Liu, Weidong Wang, Shenglin Luo*, Rong Li**

T. Li, M. Gao, Z. Wu, J. Liu, S. Luo, R. Li.
Institute of Combined Injury, State Key Laboratory of Trauma, Burns and Combined Injury, Chongqing Engineering Research Center for Nanomedicine, College of Preventive Medicine, Third Military Medical University (Army Medical University), Chongqing 400038, China
E-mail: luosl@tmmu.edu.cn; lrong361@126.com

T. Li, J. Yang, X. Gong
Center for Joint Surgery, Southwest Hospital, Third Military Medical University (Army Medical University), Chongqing 400038, China

M. Gao, Z. Wu, W. Wang
Department of Radiation Oncology, Sichuan Cancer Hospital, Sichuan Key Laboratory of Radiation Oncology, School of Medicine, University of Electronic Science and Technology of China, Chengdu, Sichuan 610041, China
E-mail: wwdwyl@uestc.edu.cn

B. Mo, S. Yu
Department of Oncology, Southwest Hospital, Third Military Medical University (Army Medical University), Chongqing 400038, China

Materials and methods

Materials

$ZrOCl_2·8H_2O$ (Zr_6 clusters), tetrakis(4-carboxyphenyl)porphyrin (TCPP), and dimethylformamide (DMF) were purchased from Aladdin (Shanghai, China). $TaCl_5$ was obtained from Sigma-Aldrich (St. Louis, MO, USA). An annexin V–fluorescein isothiocyanate (FITC)/propidium iodide (PI) apoptosis kit was obtained from MedChem Express (NJ, USA). Terminal deoxynucleotidyl transferase dUTP nick-end labeling (TUNEL) solution was purchased from Beyotime Biotechnology (Shanghai, China). All other reagents were obtained from commercial sources and directly used without further purification.

Synthesis of Zr–porphyrinic metal–organic frameworks

Zr–porphyrinic metal–organic frameworks (ZM) were synthesized using the solvothermal method as previously described. [1] Briefly, $ZrOCl_2·8H_2O$ (150 mg), TCPP (50 mg), and benzoic acid (1.4 g) were dissolved in 50 mL of DMF solution under ultrasonic conditions. The mixed solution was placed in a preheated oil bath at 90°C and vigorously stirred for 5 h. After cooling, the crude product was collected by centrifugation at 15,000 *g* for 10 min and sequentially washed three times with DMF and ethanol. The ZM product was dispersed in DMF for further use.

Preparation of TZM

$TaCl_5$ (100 mg) was dispersed in 10 mL of ZM (1 mg/mL) solution. The reaction mixture was added to a high-pressure reaction kettle and then incubated in a muffle furnace at 200°C for 24 h. After cooling, the crude product was collected by centrifugation at 15,000 *g* for 10 min and sequentially washed three times with DMF and ethanol. Finally, the TZM product was obtained by centrifugation at 1,000 g for 5 min to remove large particles and then stored at 4°C for further use.

Characterization

After being dried at room temperature and sputter-coated with gold, the TZM solution was deposited onto a silicon slide and then observed via scanning electron microscopy (SEM) using a Hitachi S-3400N SEM (Hitachi, Japan) at an accelerating voltage of 2 kV. Transmission electron microscopy (TEM) and elemental mapping were obtained using a JEM 2100F (JEOL, Japan) instrument with a molybdenum net at an accelerating voltage of 200 kV. The particle size and zeta potential were analyzed using a Malvern Zetasizer Nano ZS instrument (Malvern, UK). Ultraviolet (UV) visible absorption spectra and fluorescence spectra were recorded using a UV spectrophotometer UV-3600 (Shimadzu, Japan) and a near-infrared (NIR) fluorescence spectrometer (Thermo Fisher Scientific, USA), respectively. X-ray diffraction was performed using an X-ray diffractometer (D8 ADVANCE, Bruker, Germany) at the 2θ range of 5°–60°. The valence values of Ta and Zr were determined through X-ray photoelectron spectroscopy using a 250Xi X-ray photoelectron spectrometer (Thermo Fisher Scientific). Electron spin resonance spectroscopy was performed on a Bruker A300-10 electron paramagnetic resonance spectrometer. NIR fluorescence imaging was performed using the Pearl Trilogy *in vivo* imaging system (LI-COR, USA). Computed tomography (CT) and photoacoustic (PA) imaging were conducted using a SkyScan1276 MicoCT system (Bruker) and a LOIS-3D PA imaging system (TomoWave Laboratories, USA), respectively. Confocal fluorescence images were obtained through confocal laser-scanning microscopy (CLSM) using a Leica TCS-SP5 confocal system (Leica, Germany). Flow cytometry was performed using a FACSVerse flow cytometer (BD Biosciences).

Extracellular $^1O_2$, reactive oxygen species (ROS), and •OH generation

For the detection of extracellular $^1O_2$ generation, deionized water, ZM, and TZM (100 μg/mL) were pre-mixed with the probe Singlet Oxygen Sensor Green (2 μM) in 2% methanol aqueous solution. The mixtures were irradiated with X-ray at different doses (0, 2, 4, 6, and 10 Gy), and the fluorescence intensity (λ_{ex} = 504 nm; λ_{em} = 525

nm) was detected using a fluorescence spectrometer.

The production of ROS in the solution was measured using the ROS probe 2′,7′-dichlorodihydrofluorescein diacetate (H_2DCF-DA, Beyotime). Briefly, 0.5 mL of dimethyl sulfoxide with 12.5 μL of H_2DCF-DA (10 mM) was mixed with 2 mL of 0.01 M NaOH under dark conditions, and then the solution was stirred for 0.5 h at room temperature. The reaction was stopped by adding 10 mL of phosphate buffer saline (PBS; 25 mM, pH 6.50) to the solution, finally producing the reagent for ROS detection (H_2DCF, 10 μM). Deionized water, ZM, and TZM (100 μg/mL) were pre-mixed with an equivalent solution of H_2DCF. After irradiation with or without X-ray (6 Gy), the fluorescence intensity of DCF (λ_{ex} = 488 nm; λ_{em} = 530 nm) in the final solution was measured using a fluorescence spectrophotometer. The generation of •OH was further detected using the •OH-specific indicator disodium terephthalate (TA), which can capture •OH to form the fluorescent product disodium 2-hydroxy terephthalate (TAOH). [2] Briefly, a TA solution (5 mM) was added to different groups. After irradiation with or without X-ray (6 Gy), the fluorescence intensity of TAOH (λ_{ex} = 315 nm; λ_{em} = 435 nm) in the final solution was recorded using a fluorescence spectrophotometer.

Computational methods

The geometries of the ground-state structures of ZM and TZM were fully optimized without any structural constraints. Density functional theory (DFT) with the B3LYP functional, including the D3 version of Grimme dispersion, was employed. Double-zeta quality basis sets with LANL2DZ for Zr and 6-31g(d) for other atoms (Ta) were adopted in the calculations. All calculations were performed using the Gaussian 09 package. Time-dependent DFT calculations were performed to determine the vertical excitation energies for 10 singlet and 10 triplet excited states of TCPP, ZM, and TZM.

Cell culture

Mouse osteosarcoma K7M2 cancer cells were purchased from the American Type Culture Collection (ATCC, Manassas, USA) and cultured in ATCC-recommended medium (Hyclone, Utah, USA) supplemented with 10% fetal bovine serum (FBS, γ-irradiated, sterile-filtered, suitable for cell culture and hybridoma) and 1% penicillin and streptomycin (Beyotime, Shanghai, China). The cells were incubated under 5% CO_2 at 37°C, passaged regularly, and then sub-cultured to ~80%/90% confluence before all the experiments.

Cell viability evaluation

The cytotoxicity of ZM and TZM against K7M2 cells was examined using the standard Cell Counting Kit-8 (CCK-8) assay. Briefly, cells were seeded into 96-well plates at a density of 5×10^3 cells/well and then treated with gradient concentrations (0, 3.125, 6.25, 12.5, 25, 50, 100, and 200 μg/mL) of ZM or TZM for 24 h. The cells were exposed to a series of radiation doses (0, 2, 4, 6, and 10 Gy) after treatment with ZM or TZM to evaluate the efficacy of RT-RDT. CCK-8 working solution (10 μL per well) was added and incubated for another 2 h. Finally, the absorbance was obtained at 450 nm using a multiplate reader (1510, Thermo Fisher Scientific). U2OS cells (ATCC) were exposed to a series of radiation doses (0, 2, 4, 6, and 10 Gy) after treatment with ZM or TZM to confirm the radiosensitizing effect of TZM on other cell lines.

Cell uptake

K7M2 cells (5×10^4 per dish) were plated in 35 mm^3 culture dishes and cultured for another 12 h. Subsequently, the cells were incubated with TZM (100 μg/mL) for different durations (1, 2, 4, 6, 12, 24, and 36 h). Cell nuclei were stained with 4′,6-diamidino-2-phenylindole and imaged using a fluorescence microscope.

Colony formation assay

K7M2 cells were divided into six groups: Control, ZM, TZM, X-ray (6 Gy), ZM

+ X-ray (6 Gy), and TZM + X-ray (6 Gy). After receiving the desired treatment, cells (800 cells/well) were inoculated into six-well plates and then cultured under standard conditions. The cell medium was refreshed once every 3 days until obvious colonies formed in the control group. Subsequently, colonies were fixed with 4% paraformaldehyde for 30 min and stained with crystal violet for 20 min. Cells were photographed, and the number of colonies (more than 50 cells) was counted using ImageJ.

Cell apoptosis analysis

K7M2 cells (3×10^5 per well) were plated on 60 mm culture dishes overnight and treated with PBS, TZM, X-ray (6 Gy), or TZM + X-ray. The cells were co-stained using an Annexin V-FITC/PI apoptosis kit in accordance with the manufacturer's instructions and then subjected to flow cytometry. For TUNEL staining, cells were fixed with 4% paraformaldehyde for 30 min and permeabilized with 0.3% Triton X-100 for 5 min at room temperature. The cells were then co-incubated with 100 μL of TUNEL solution for 60 min at 37°C in the dark. TUNEL-positive cells were observed and photographed using a fluorescence microscope.

DNA damage evaluation

Cellular DNA damage was evaluated using the comet assay and immunofluorescence staining for γH2AX in accordance with our previous methods. [3]

Intracellular ROS and $^1O_2$ generation

K7M2 cells (2×10^5 per well) were seeded into six-well plates and treated with TZM for 12 h. Then, the cells were subjected to X-ray irradiation (6 Gy) and incubated for another 2 h. After washing with PBS, the cells were stained with DCFH-DA and BBoxiProbe® O66 probes following the manufacturer's instructions. The cells were imaged using a fluorescence microscope (X51; Olympus, Tokyo, Japan).

Immunogenic cell death and dendritic cell (DC) maturation *in vitro*

K7M2 cells (5×10^4 per dish) were plated in 35 mm^3 culture dishes and treated with PBS, TZM (100 μg/mL), X-ray irradiation (6 Gy), or TZM + X-ray. The cells were subjected to immunofluorescent staining for calreticulin exposure and high-mobility group box 1 (HMGB1) release detection as previously described. [5] Finally, fluorescence images were captured using CLSM and analyzed using ImageJ. For DC maturation evaluation, bone marrow-derived cells were isolated from BALB/c mice and cultured in accordance with standard protocols. [6] The release of HMGB1 in the supernatant was detected using an enzyme-linked immunosorbent assay kit (Bioswamp, Wuhan, China) in accordance with the manufacturer's instructions. DCs were incubated with K7M2 cell supernatants from the various groups for 12 h in a Transwell co-culture system. The cells were then stained with antigen-presenting cell (APC) anti-mouse CD80 and PE-Cy7 anti-mouse CD86 antibodies for flow cytometry.

CT/PA imaging properties of TZM in solutions

For *in vitro* CT imaging, TZM at different concentrations (0, 1, 5, 10, and 30 mg/mL) was scanned using a CT imaging instrument. For PA imaging, TZM was dissolved in an aqueous solution at various concentrations (0, 1, 5, 10, and 30 mg/mL) and then transferred into individual tube phantoms. The PA contrasting capacity of TZM was evaluated using an NIR PA imaging system excited by an 808 nm NIR laser.

***In vivo* NIR/CT/PA multimodal imaging**

Six-week-old female BALB/c mice (18–22 g) were obtained from the Laboratory Animal Center of the Third Military Medical University (Chongqing, China). All animal experiments were approved by the Ethics Committee of the Third Military Medical University (Accreditation number, AMUWEC20213589, Chongqing, China).

A small-animal NIR imaging system was used for *in vivo* NIR imaging. TZM (20 mg/kg) was intravenously injected into female K7M2 tumor-bearing BALB/c mice, and images were captured at various time points (0–24 h). The mice were euthanized 24 h after injection, and the major organs (heart, liver, spleen, lung, kidney, muscle, and intestine) and tumor tissues were harvested for *ex vivo* NIR imaging. The relative fluorescence intensities in the tumor area and other organs were semi-quantitatively analyzed using Image Studio (Version 5.2, Image-Studio-Pro). Female K7M2 tumor-bearing BALB/c mice were immediately subjected to CT imaging after the intravenous injection of TZM or ZM (20 mg/kg). For PA imaging, K7M2 tumor-bearing BALB/c nude mice intravenously injected with TZM (20 mg/kg) were imaged at various time points using an NIR PA imaging system excited by an 808 nm NIR laser. The PA amplitude in the tumor regions was quantified using TomoView (TomoWave).

Pharmacokinetics

K7M2 tumor-bearing BALB/c mice were intravenously injected with TZM (20 mg/kg) to evaluate the blood circulation and biodistribution of TZM. Blood samples, major organs (heart, liver, spleen, lung, kidney, intestine, stomach, and muscle), and tumor tissues were harvested at different time points after the injection. Then, the Ta content in the different samples was determined using inductively coupled plasma mass spectroscopy (Agilent 7500, USA) and calculated as the percentage of injected dose per gram of tissue (ID%/g).

***In vivo* antitumor experiments**

K7M2 cells (1×10^7/mice) were subcutaneously inoculated into the left flanks of BALB/c mice to develop a unilateral subcutaneous tumor model. Tumor-bearing mice were randomly divided into four groups (n = 5 per group): PBS, TZM, X-ray, and TZM + X-ray. The mice in the TZM and TZM + X-ray groups were intravenously injected with TZM (5 mg/kg) on days 0, 2, and 4, whereas those in the X-ray and

TZM + X-ray groups received X-ray irradiation (6 Gy) on days 1, 3, and 5 after TZM injection for 24 h. The tumor size (length (a) and width (b)) and body mass of the mice were measured every 2 days for 14 days.

When the volume of the primary tumor (right flank) reached approximately 50 mm^3, K7M2 cells (1×10^6/mice) were subcutaneously inoculated into the left flank (distant tumor) of the mice to develop a bilateral subcutaneous tumor model. Tumor-bearing mice were randomly divided into four groups (n = 5 per group): PBS, anti-PD-L1 + X-ray, TZM + X-ray (6 Gy), and TZM + anti-PD-L1 + X-ray. The doses of TZM and X-ray radiation were the same as before, whereas the anti-PD-1 antibody (75 μg/mouse) was administered intravenously following X-ray irradiation. Only the primary tumors were exposed to X-ray irradiation, and all other regions were shielded using a lead cover. Tumor volume was calculated using the following formula:

$$V = 1 / 2ab^2$$

On day 14, all tumor-bearing mice were euthanized, and the tumor tissues were weighed. Meanwhile, the tumor tissues were immediately fixed in 4% paraformaldehyde for further hematoxylin and eosin (H&E), Ki-67, TUNEL, and immunofluorescence staining.

When the volume of the primary tumor reached approximately 100 mm^3, K7M2 cells (1×10^6/mice) were intravenously implanted into the mice to develop a lung metastasis model. The mice were administered the same treatments as those used to establish the bilateral tumor model, and the mice were euthanized on day 21. The representative lungs of each group of mice were photographed and fixed in 4% paraformaldehyde for further H&E staining.

Flow cytometry of immune cells

Tumor-draining lymph nodes and tumor tissues were harvested 3 days after the last administration. DCs and tumor-infiltrating lymphocytes were isolated according to standard protocols. For DCs, the cell suspension was co-stained with FITC anti-mouse Lineage, APC anti-mouse CD80 antibody, and BV421 anti-mouse CD11c

antibody. For T cells, the cell suspension was stained with APC anti-mouse CD45, FITC anti-mouse CD3, and BV510 anti-mouse CD8 antibodies. The percentages of mature DCs and tumor-infiltrating $CD8^+$ T cells were quantified using flow cytometry.

Western blot

K7M2 cells were treated with PBS, TZM (100 μg/mL), X-ray irradiation (6 Gy), or TZM + X-ray for the desired times. Total proteins were isolated from cells using radioimmunoprecipitation assay lysis and quantified using a bicinchoninic acid protein kit in accordance with the manufacturer's instructions. The expression levels of cGAS-STING-TBK1-IRF3 signaling-related genes were examined by western blot as previously reported. [4] After incubation with the indicated primary and secondary antibodies, blot images were obtained and visualized using the Amersham Imager 600 System (GE, Pittsburgh, USA) after enhanced chemiluminescence staining, and relative gene expression levels were quantified using ImageJ. Three independent experiments were performed. The following primary antibodies were used in these experiments: anti-GAPDH antibody (ab9484, Abcam), anti-cGAS antibody (ab252416, Abcam), anti-STING antibody (ab288157, Abcam), phospho-TMEM173/STING (Ser366) (AF7416, Affinity), anti-NAK/TBK1 antibody (ab40676, Abcam), phospho-TBK1 (Ser172) antibody (AF8190, Affinity), anti-IRF3 antibody (ab68481, Abcam), phospho-IRF3 (Ser396) antibody (AF2436, Affinity), and anti-PD-L1 antibody (ab213480, Abcam).

Hemolysis evaluation

Hemocompatibility of TZM was analyzed as previously described by Zhao et al. [7]. Specifically, blood samples were collected from healthy BALB/c mice, and fibrinogen was removed via repeated centrifugation (1,000 *g*, 10 min). Then, 900 μL of deionized water (positive control), PBS (pH 7.4) (negative control), and gradient TZM concentrations (0, 50, 100, 200, and 500 μg/mL) were homogenized with

defibrinated erythrocyte suspension (100 μL) by pipette mixing. Following incubation at 37℃ for 2 h, the mixture was centrifuged at 1,000 *g* for 10 min to collect the supernatant. Finally, absorbance was recorded at 540 nm using a multiplate reader (1510, Thermo Fisher Scientific), and the hemolysis ratio was calculated using the following formula:

$$\text{Hemolysis (\%)} = [(OD_{sample} - OD_{negative}) / (OD_{positive} - OD_{negative})] \times 100\%.$$

Biosafety analysis

TZM (20 mg/kg) was intravenously injected into healthy BALB/c mice, and blood samples were collected via eyeball extirpation on days 1, 7, and 14 post-injection. Hematological parameters (WBC, white blood cell; RBC, red blood cell; HGB, hemoglobin; MCV, mean corpuscular volume; MCH, mean corpuscular hemoglobin; MCHC, mean corpuscular hemoglobin concentration; RDW-CV, coefficient of variation of RBC distribution width; HCT, hematocrit) and blood biochemical parameters (ALT, alanine aminotransferase; AST, aspartate transaminase; CK, creatine kinase; LDH, lactate dehydrogenase; blood urea nitrogen, UREA; CREA, Creatinine) were measured using an automatic biochemical analyzer. Additionally, the major organs (heart, liver, spleen, lung, and kidney) were dissected and fixed in 4% paraformaldehyde for further H&E staining.

Statistical analysis

All quantitative data are expressed as the mean ± standard deviation of at least three independent biological replicates. Origin (version 2019b, MA, USA) and GraphPad Prism (version 8.3.0, CA, USA) were used to process, analyze, and graph the collected data. Two different groups were compared using independent sample Student’s *t*-test, and multiple group comparisons were performed using one-way ANOVA with Tukey’s post-hoc test. Statistical significance was considered at $p < 0.05$.

References

[1] S. Wang, Y. Chen, S. Wang, P. Li, C. A. Mirkin, O. K. Farha, *J. Am. Chem. Soc.* **2019**, 141, 2215.

[2] R. Zhou, L. Yan, X. Dong, S. Zhu, K. Chen, Y. Wu, H. Xiang, L. Li, G. Zhang, Z. Gu, Y. Zhao, *Nano Today* **2021**, 36, 101003.

[3] M. Gao, X. Huang, Z. Wu, L. Wang, S. Yuan, Z. Du, S. Luo, R. Li, W. Wang, *Materials Today. Bio* **2022**, 15, 100316.

[4] T. Li, J. Yang, C. Weng, P. Liu, Y. Huang, S. Meng, R. Li, L. Yang, C. Chen, X. Gong, *Int. J. Biol. Macromol.* **2021**, 170, 469.

[5] S. Luo, X. Luo, X. Wang, L. Li, H. Liu, B. Mo, H. Gan, W. Sun, L. Wang, H. Liang, S. Yu, *Small* **2022**, e2201298.

[6] K. Roney, *Methods Mol. Biol.* **2013**, 1031, 71.

[7] X. Guo, F. Liu, J. Deng, P. Dai, Y. Qin, Z. Li, B. Wang, A. Fan, Z. Wang, Y. Zhao, *ACS Nano* **2020**, 14, 14715.

Supplementary Figures and Table

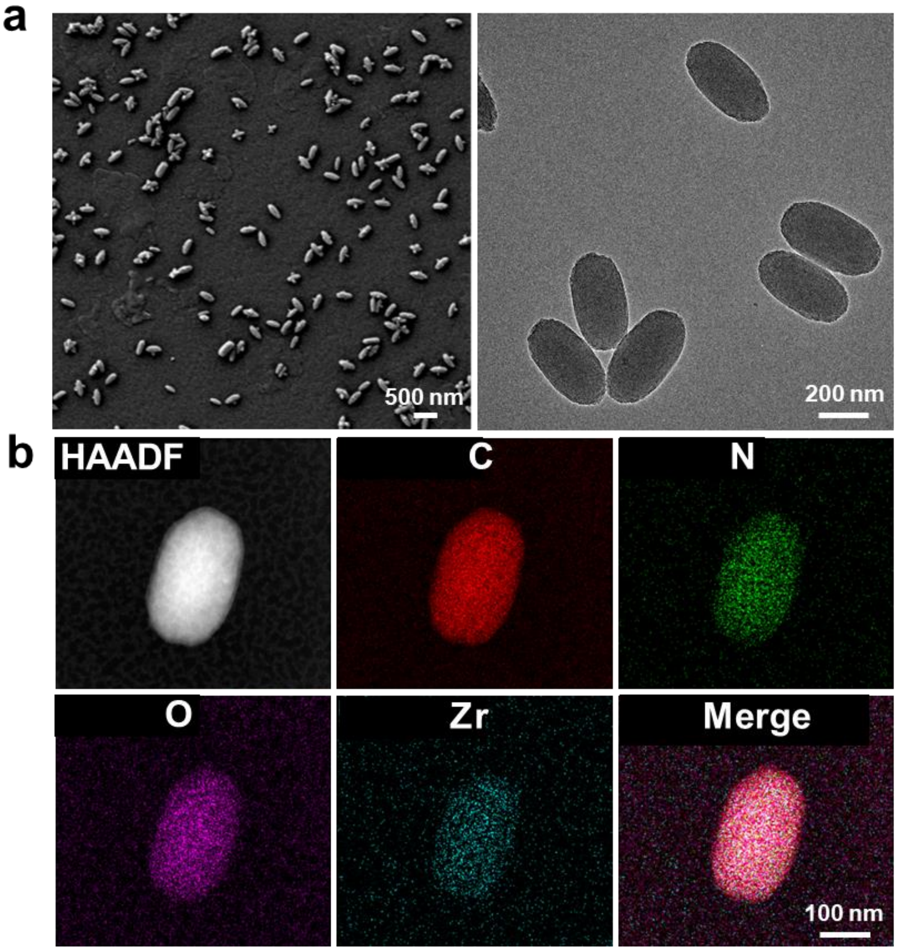

Figure S1. (a) SEM and TEM images of ZM, (b) Dark-field TEM image of ZM and corresponding TEM elemental mappings.

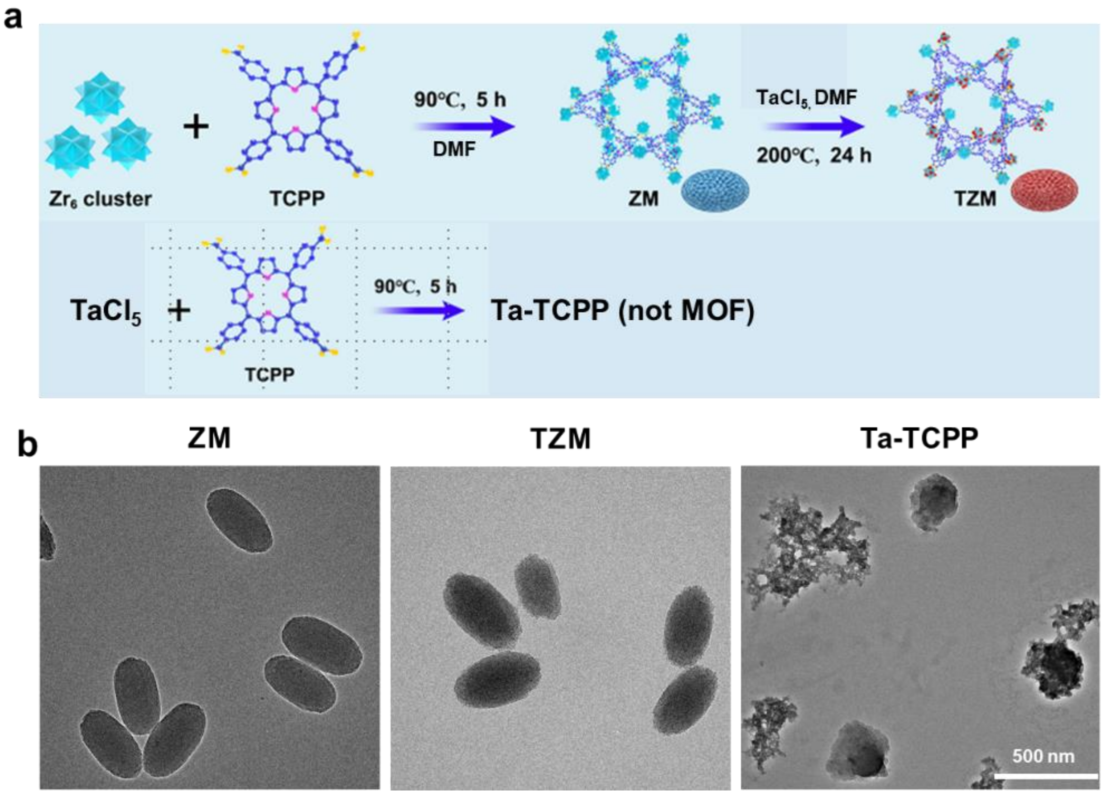

Figure S2. (a) Schematic of the preparation of TZM and Ta-TCPP. Ta and TCPP could not form a regular morphology (not MOF). (b) TEM images of ZM, TZM, and Ta-TCPP.

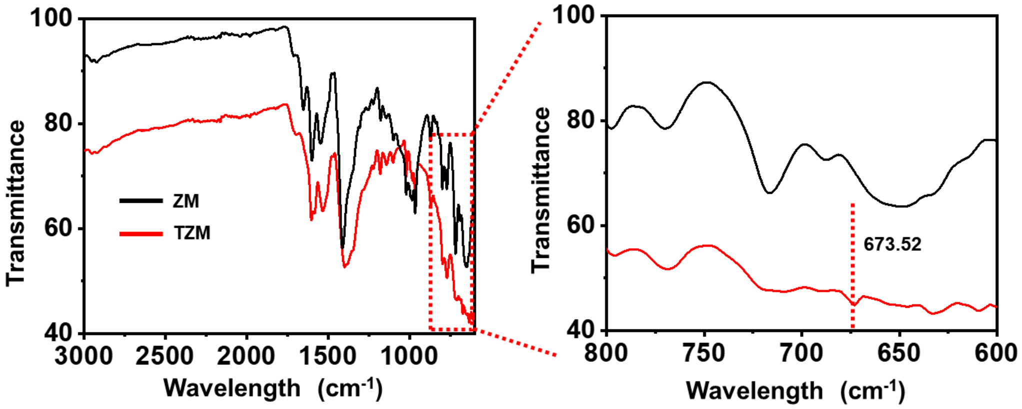

Figure S3. FT-IR spectra of ZM and TZM.

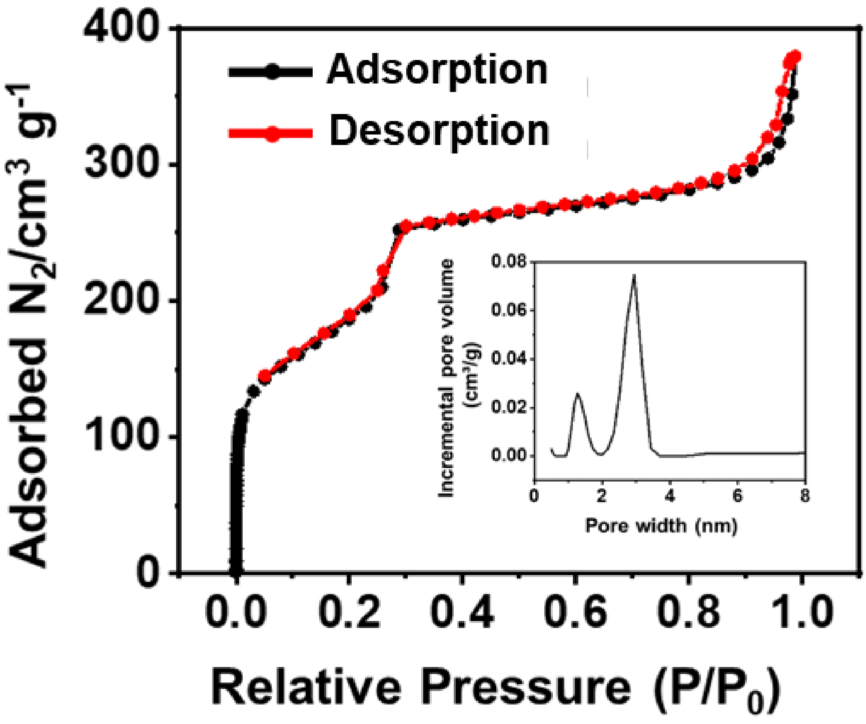

Figure S4. N_2 adsorption–desorption isotherms and DFT pore size distribution of ZM.

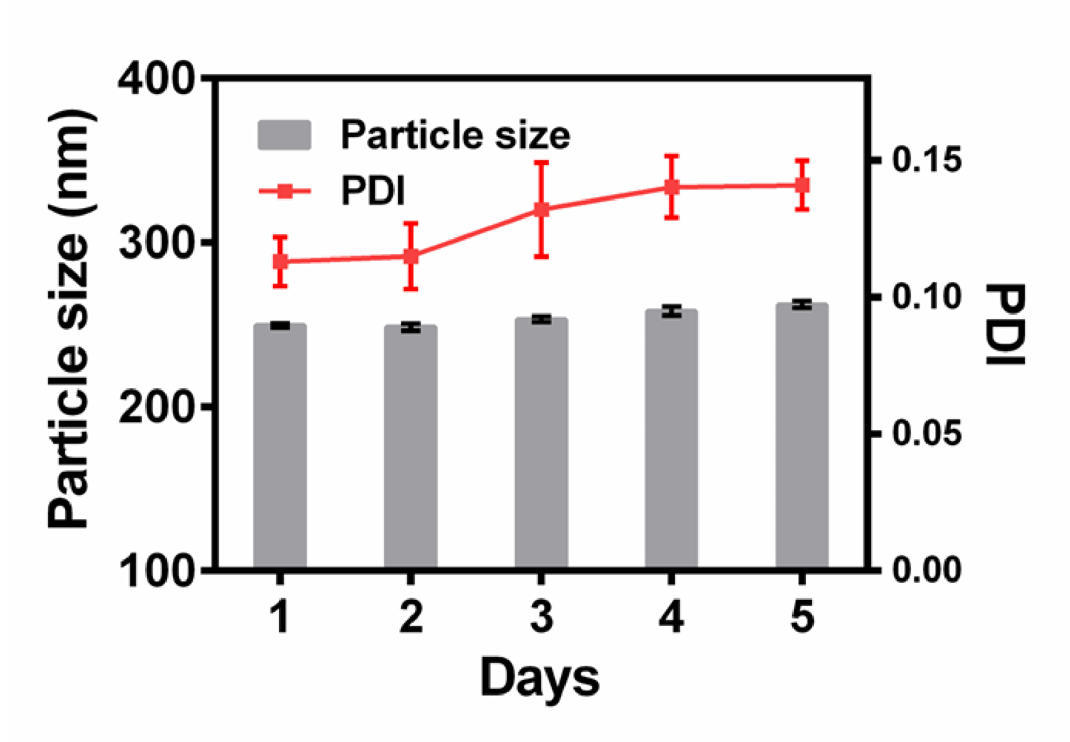

Figure S5. Hydrodynamic diameters and polydispersity index (PDI) of TZM in H_2O measured by DLS over 5 days. Data are presented as mean ± standard deviation, n = 3.

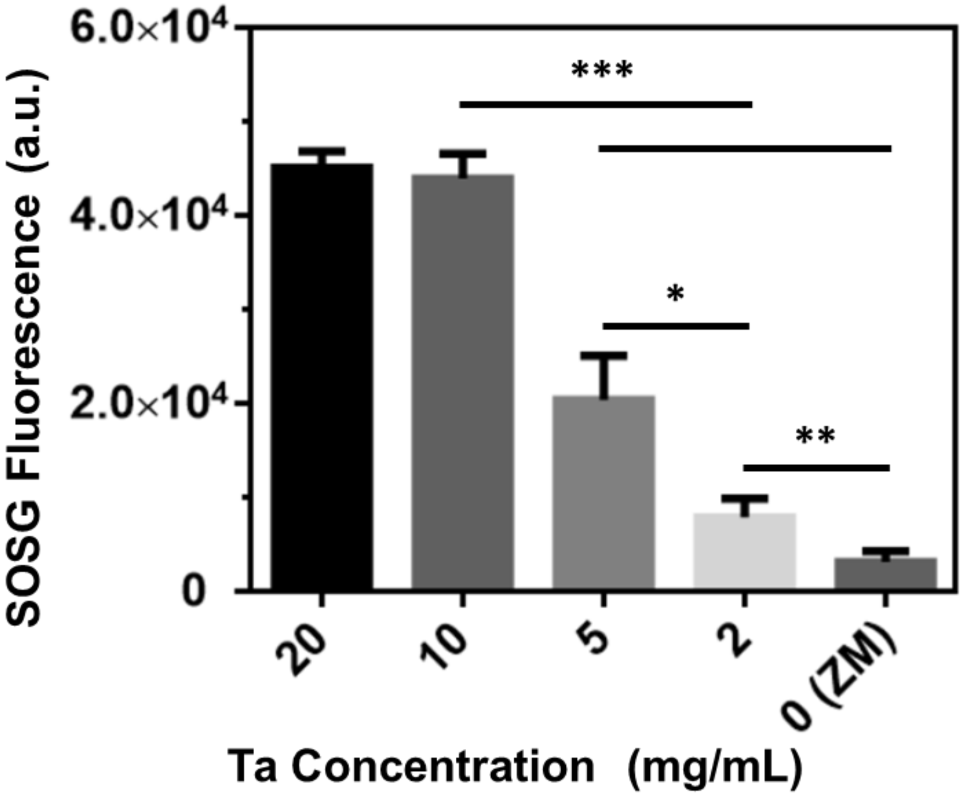

Figure S6. Fluorescence intensity of SOSG for detecting TZM-triggered $^1O_2$ after 6 Gy X-ray irradiation with different Ta concentrations. Data are presented as mean ± standard deviation, n = 3. Statistical analysis was performed using one-way ANOVA with Tukey's post-hoc test, *p < 0.05, **p < 0.01, ***p < 0.001.

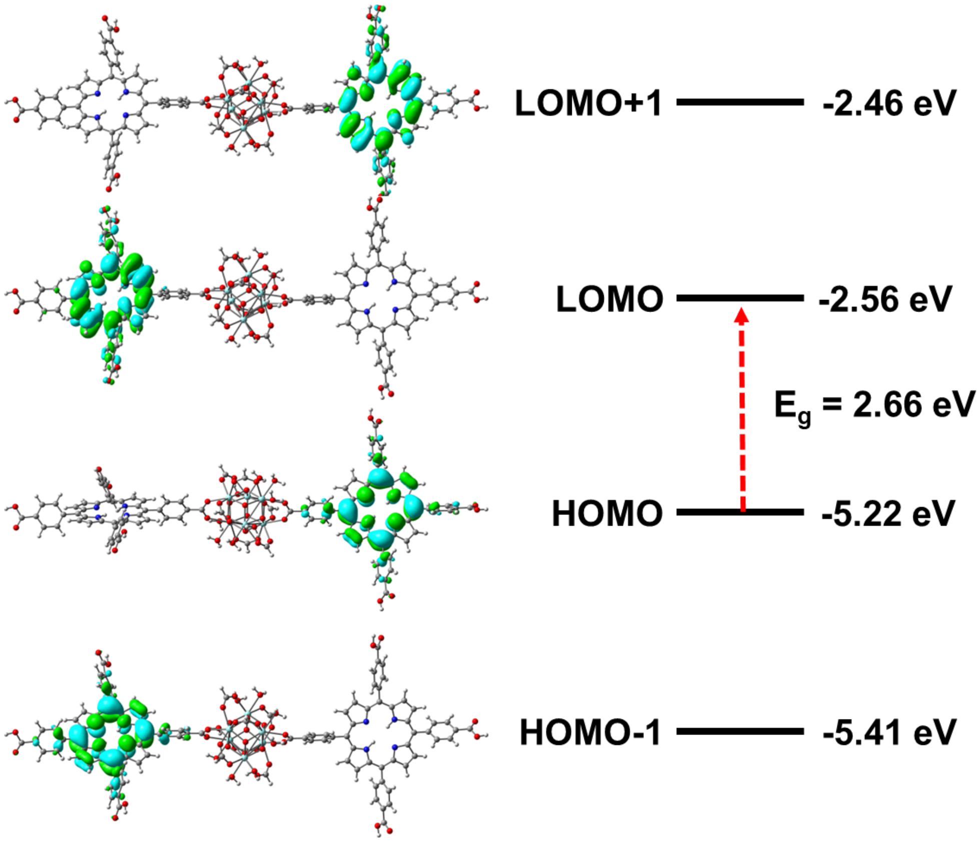

Figure S7. DFT calculation of frontier molecular orbitals and corresponding energy levels of ZM.

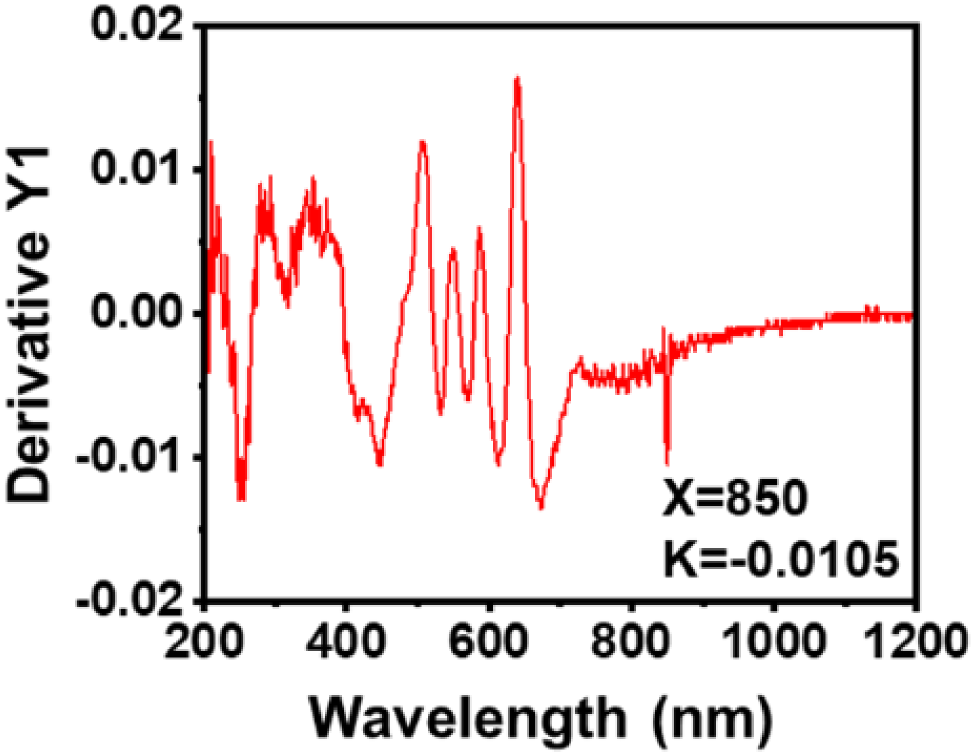

Figure S8. First derivative spectra corresponding to UV–Vis diffuse reflectance spectrum of TZM.

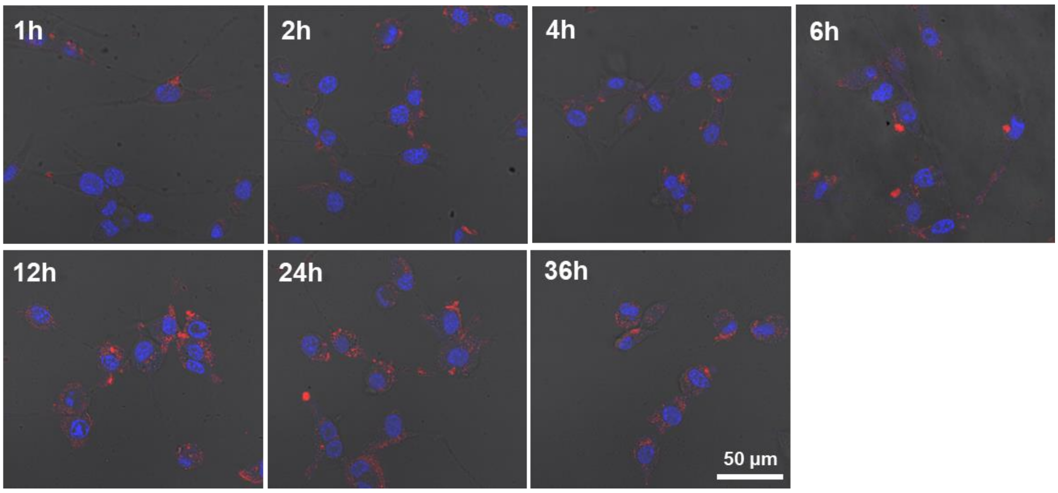

Figure S9. Fluorescence images for the cellular uptake of TZM at different time points.

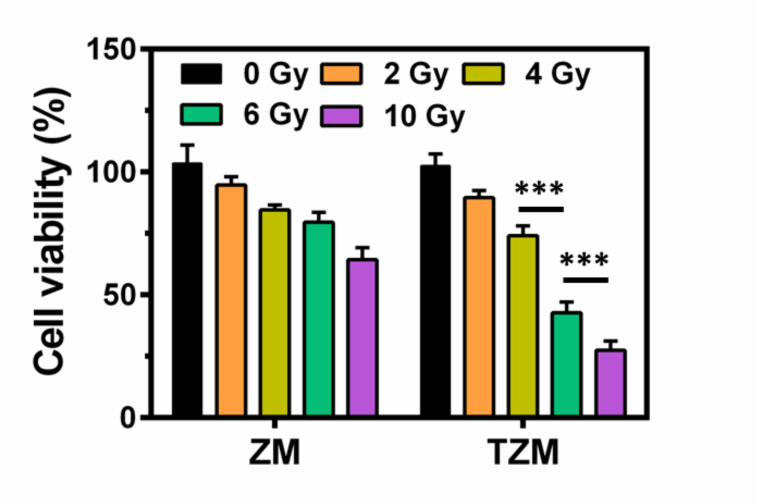

Figure S10. Relative viability of U2OS cells after treatment with ZM and TZM under different X-ray irradiation doses. Data are presented as mean ± standard deviation, n = 3. Statistical analysis was performed using one-way ANOVA with Tukey's post-hoc test, *$p < 0.05$ and ***$p < 0.001$.

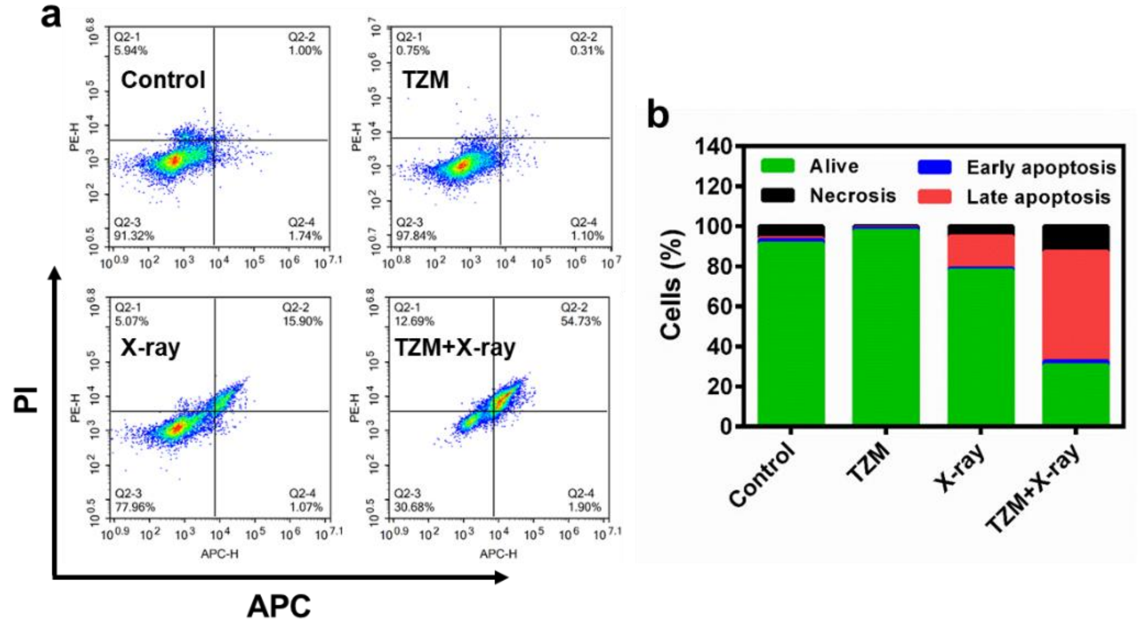

Figure S11. (a) Flow cytometric analysis of cell apoptosis after different treatments. (b) Percentage of cells (alive, necrosis, early apoptosis, and late apoptosis).

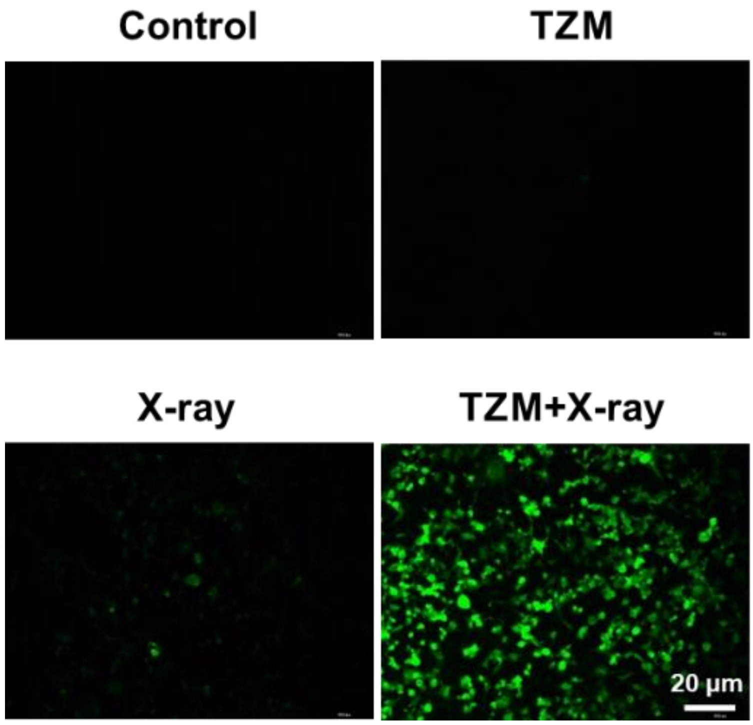

Figure S12. Fluorescence images of K7M2 cells stained with TUNEL kit after different treatments.

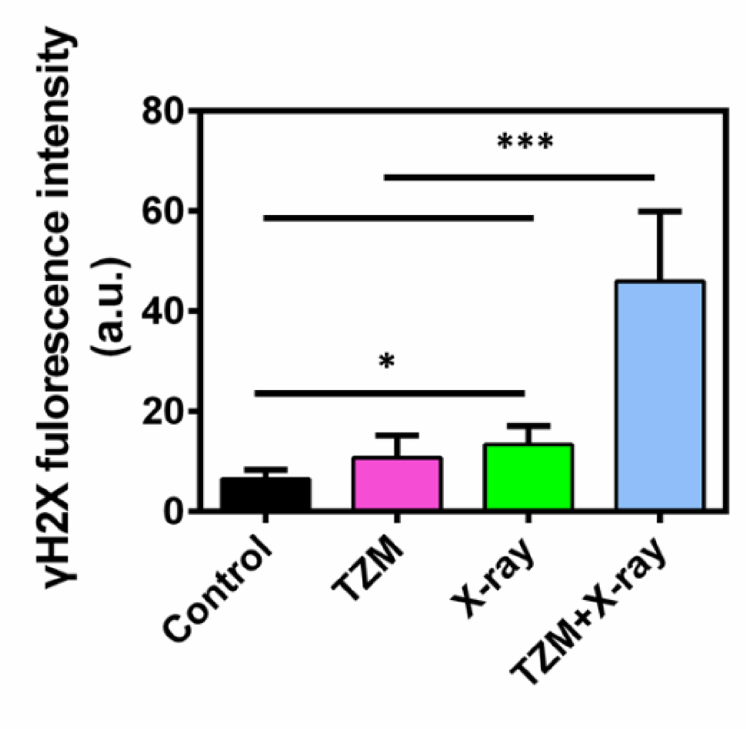

Figure S13. Semi-quantitative analysis of γH2X fluorescence image intensities in K7M2 cells based on the image displayed in Figure 3e. Data are presented as mean ± standard deviation, n = 10. Statistical analysis was performed using one-way ANOVA with Tukey's post-hoc test, *$p < 0.05$ and ***$p < 0.001$.

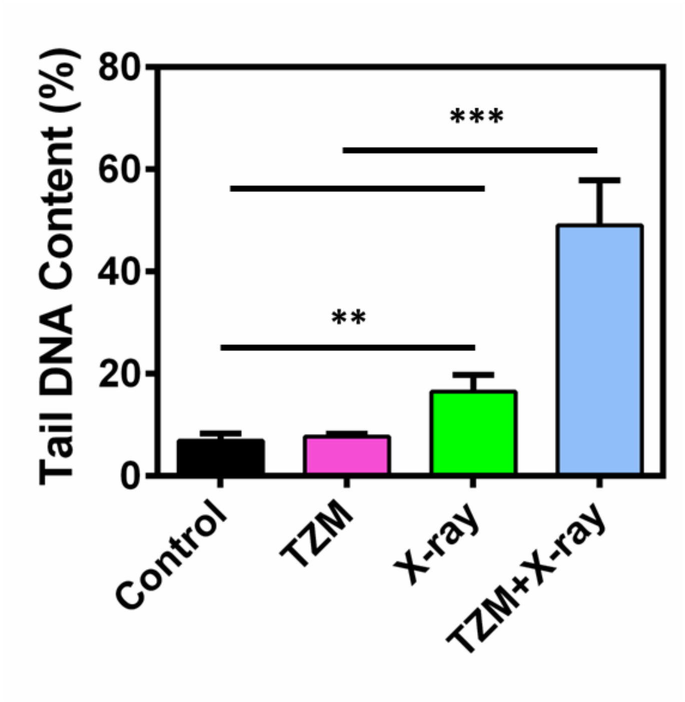

Figure S14. Semi-quantitative analysis of comet assay image intensities in K7M2 cells based on the image displayed in Figure 3f. Data are presented as mean ± standard deviation, n = 10. Statistical analysis was performed using one-way ANOVA with Tukey's post-hoc test, $^{**}p < 0.01$ and $^{***}p < 0.001$.

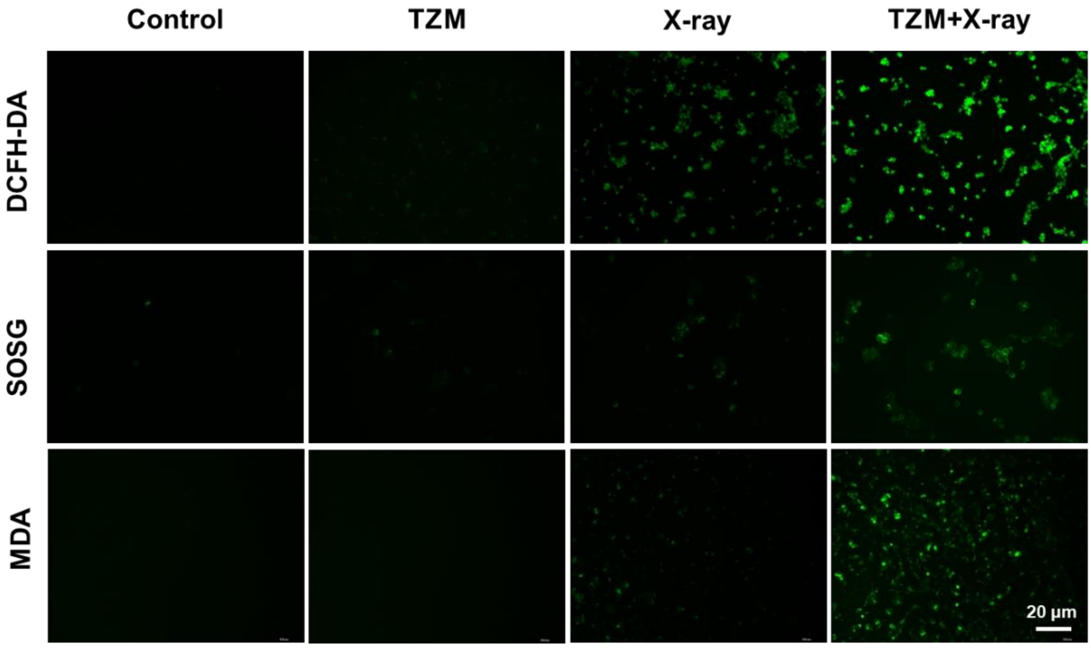

Figure S15. Fluorescence images of DCFH-DA, SOSG, and malondialdehyde (MDA) staining in K7M2 cells after different treatments.

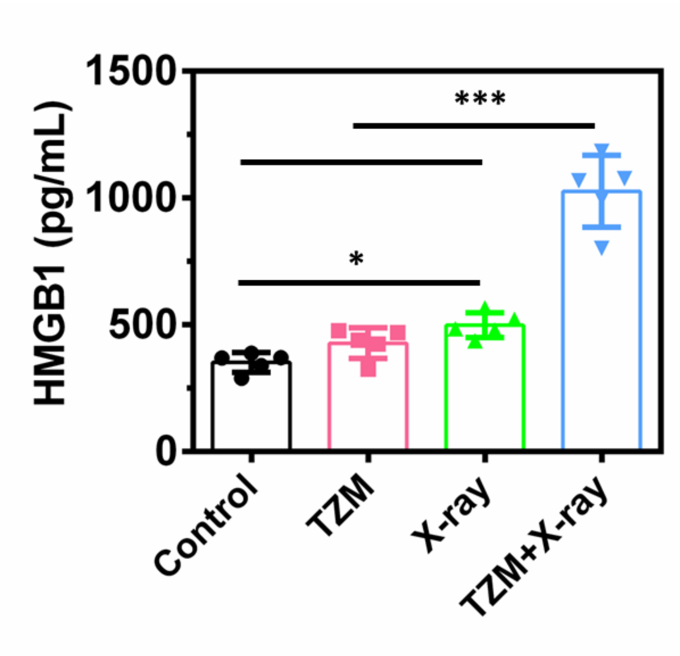

Figure S16. Level of HMGB1 in the extracellular environment was detected with ELISA kit after different treatments. Data are presented as mean ± standard deviation, n = 5. Statistical analysis was performed using one-way ANOVA with Tukey's post-hoc test, *$p < 0.05$, ***$p < 0.001$.

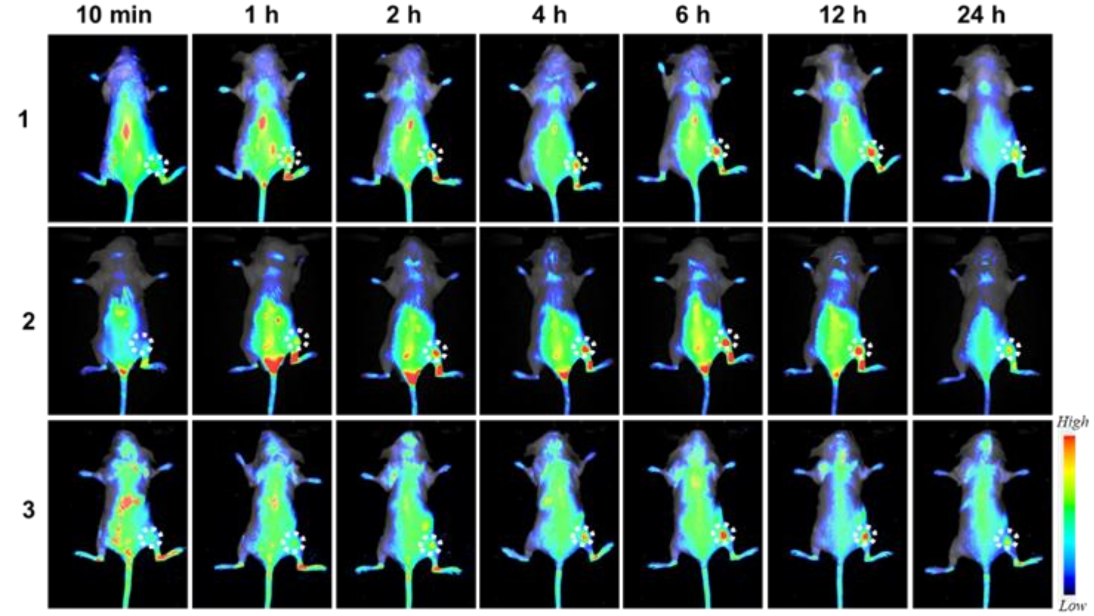

Figure S17. NIR fluorescence imaging of whole mice in triplicate.

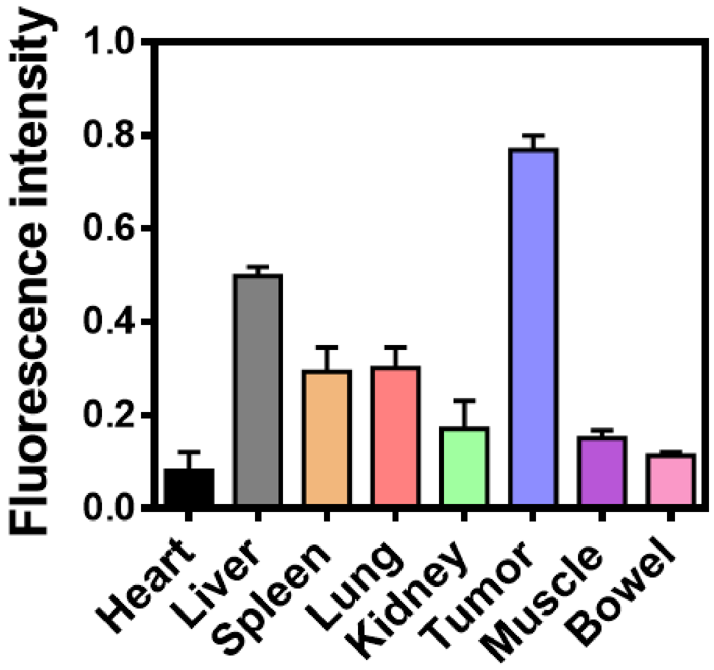

Figure S18. Semi-quantitative analysis of fluorescence intensities in organs and tumor. Data are presented as mean ± standard deviation, n = 3.

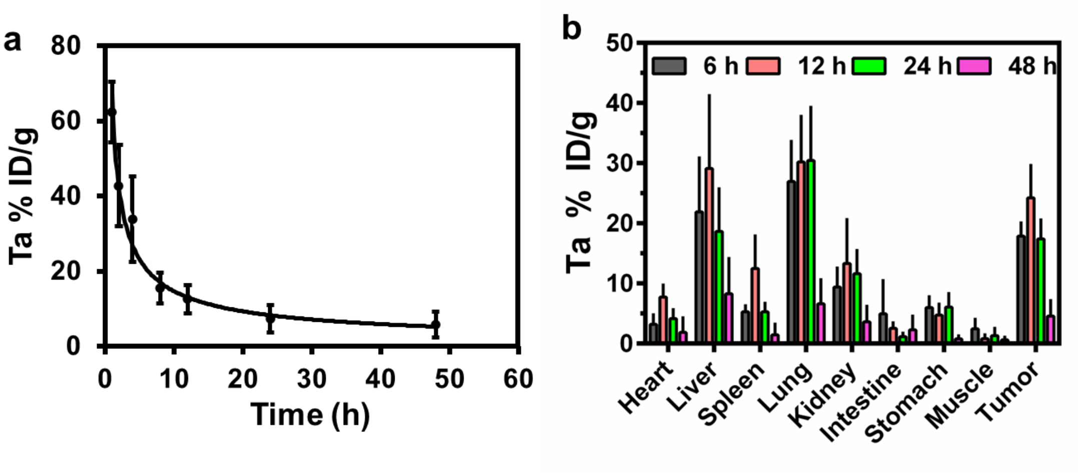

Figure S19. (a) Ta concentration in blood circulation of mice after the intravenous administration of TZM at different time points. (b) Ta biodistribution in vital organs (heart, liver, spleen, lung, kidney, intestine, stomach, and muscle) and tumor site post 6, 12, 24, and 48 h injection. Data are presented as mean ± standard deviation, n = 3.

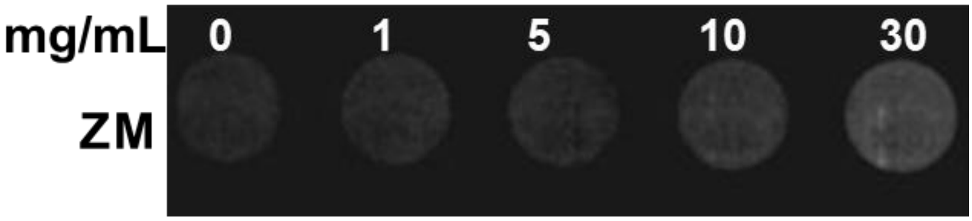

Figure S20. *In vitro* CT images of ZM at various gradient concentrations (0–30 mg/mL).

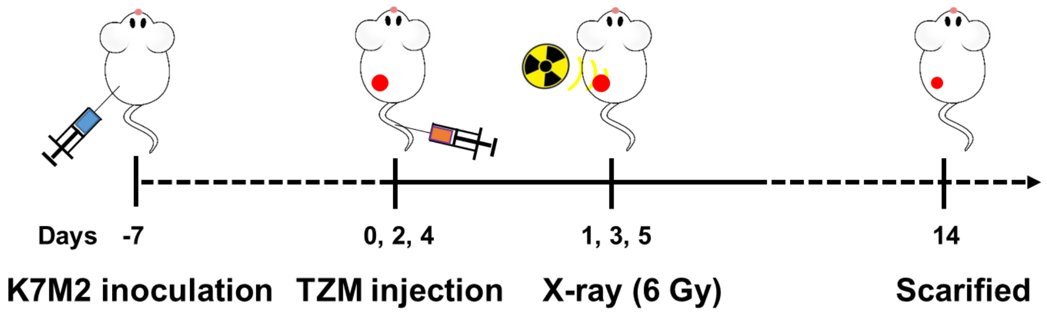

Figure S21. Schematic of the treatment of K7M2 tumor-bearing mice.

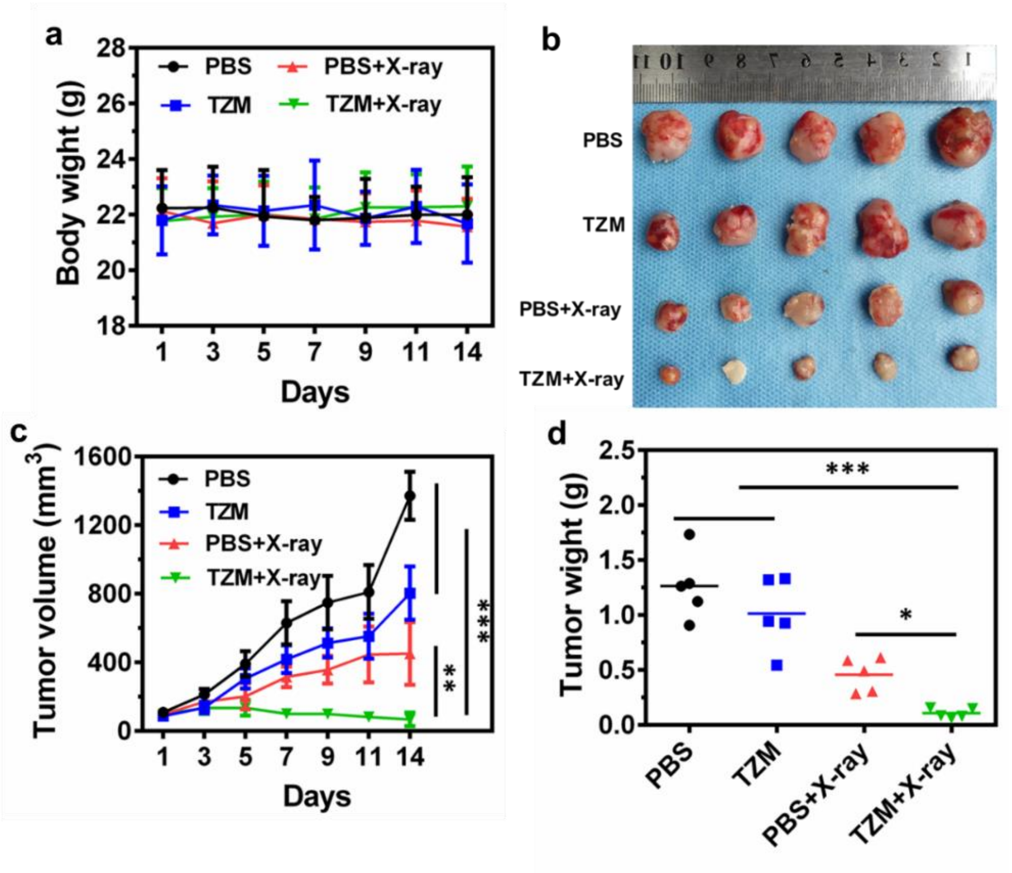

Figure S22. (a) Body weight of mice after receiving the indicated treatments for 14 days in a unilateral tumor model. (b) Digital photographs of the tumor harvested from mice at day 14 after indicated treatments. (c) Tumor volume curves after different treatments. (d) Weight of the tumor harvested from mice on day 14 after the indicated treatments. Data are presented as mean ± standard deviation, n = 5. Statistical analysis was performed using one-way ANOVA with Tukey's post-hoc test, *$p < 0.05$, **$p < 0.01$, ***$p < 0.001$.

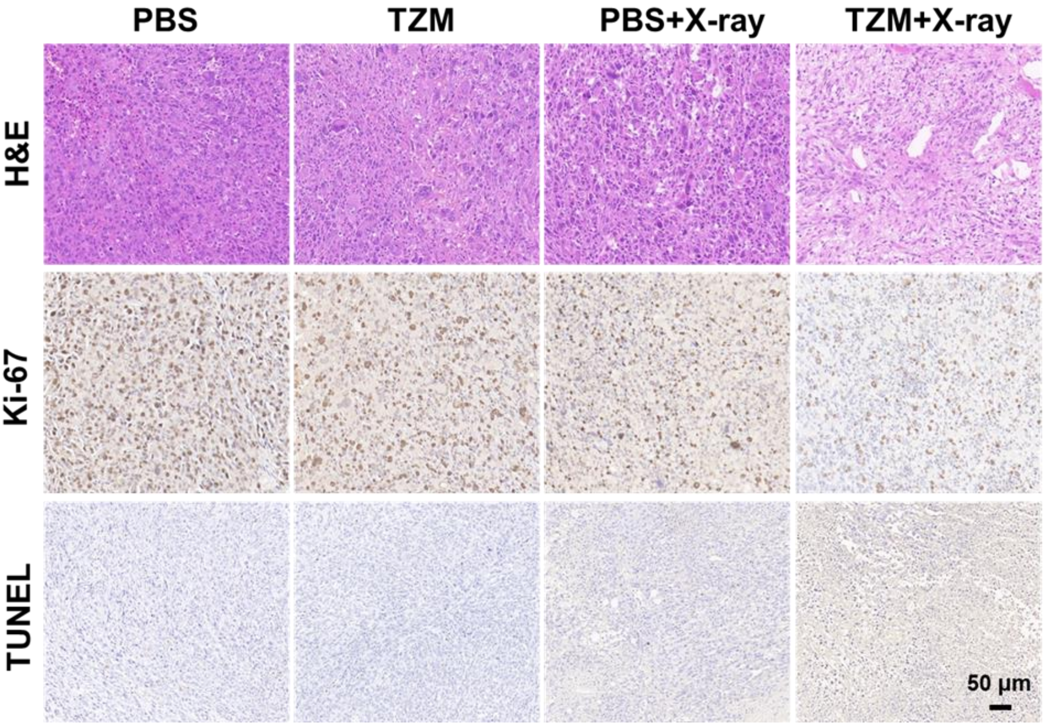

Figure S23. H&E and IHC staining (Ki-67 and TUNEL) images of single tumors on day 14 after indicated treatments.

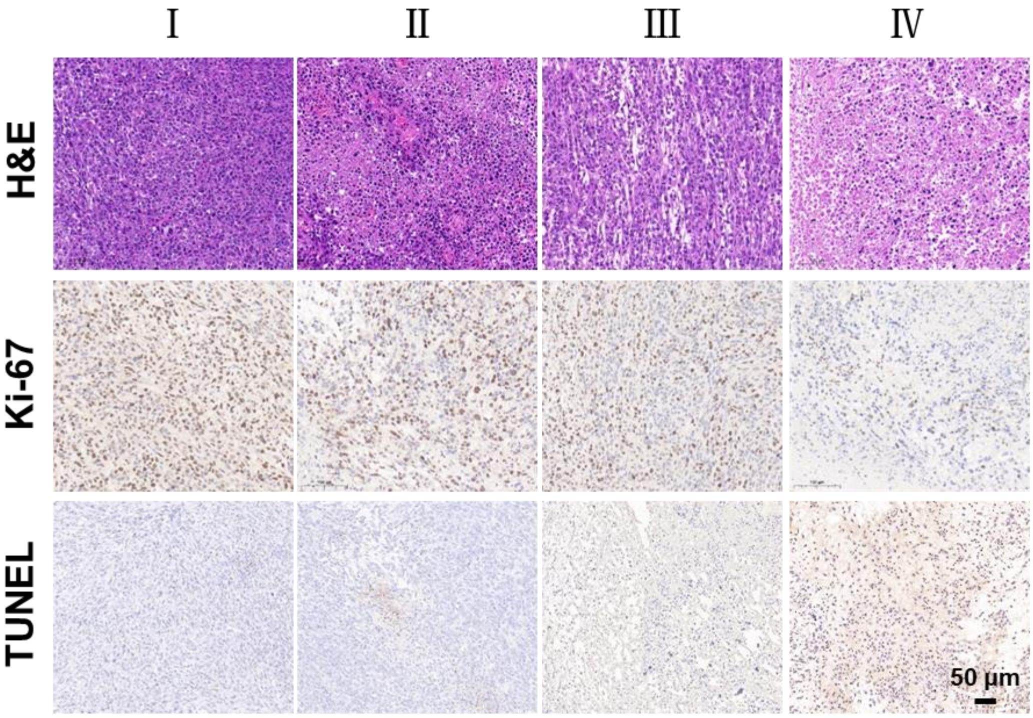

Figure S24. H&E and IHC staining (ki-67 and TUNEL) images of primary tumors at day 14 after receiving indicated treatments. For each group: I, PBS; II, anti-PD-L1+X-ray; III, TZM+X-ray; IV, TZM + X-ray + anti-PD-L1.

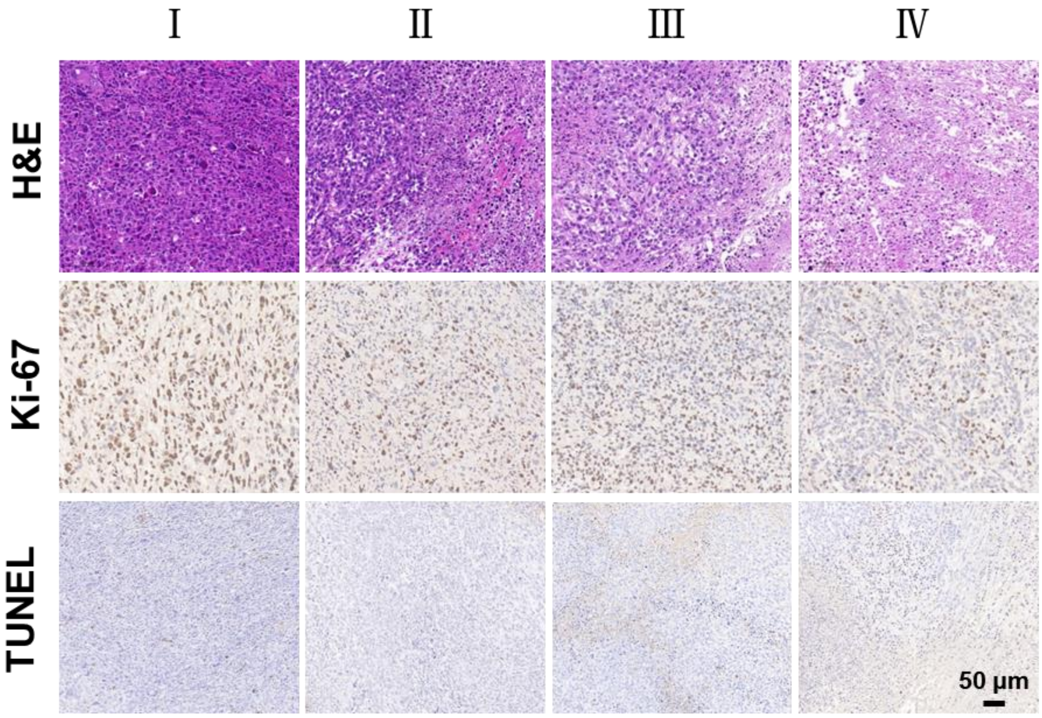

Figure S25. H&E and IHC staining (ki-67 and TUNEL) images of distant tumors at day 14 after receiving indicated treatments. For each group: I, PBS; II, anti-PD-L1+X-ray; III, TZM+X-ray; IV, TZM + X-ray + anti-PD-L1.

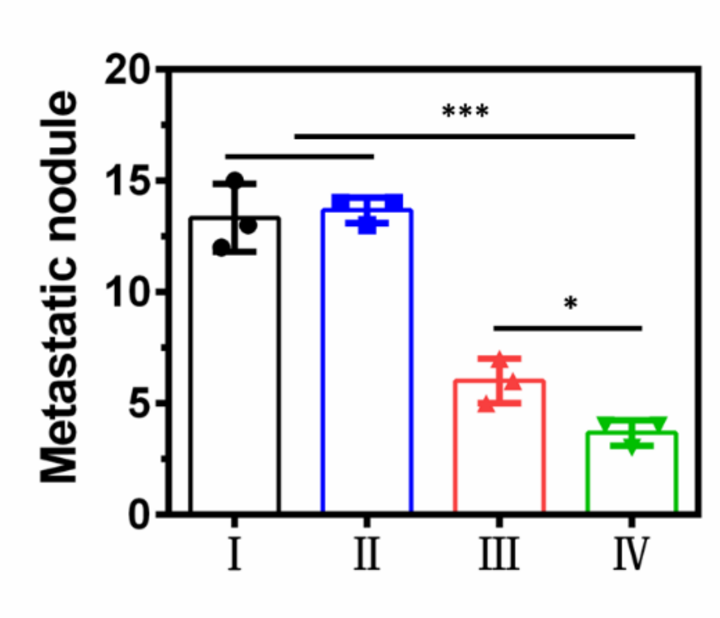

Figure S26. Calculated lung metastasis nodules of mice after receiving indicated treatments. For each group: I, PBS; II, anti-PD-L1+X-ray; III, TZM+X-ray; IV, TZM + X-ray + anti-PD-L1. Data are presented as mean ± standard deviation, n = 3. Statistical analysis was performed using one-way ANOVA with Tukey's post-hoc test, $^{*}p < 0.05$ and $^{***}p < 0.001$.

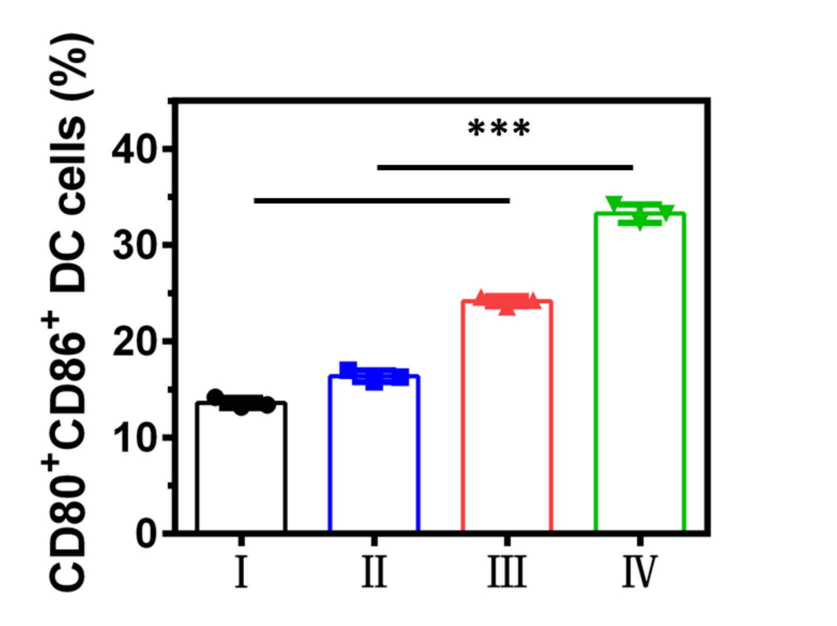

Figure S27. Percentage of mature DCs (CD80+ CD86+) in lymph nodes from tumor-bearing mice isolated on day 3 after receiving indicated treatments. For each group: I, PBS; II, anti-PD-L1+X-ray; III, TZM+X-ray; IV, TZM + X-ray + anti-PD-L1. Data are presented as mean ± standard deviation, n = 3. Statistical analysis was performed using one-way ANOVA with Tukey's post-hoc test, ***$p < 0.001$.

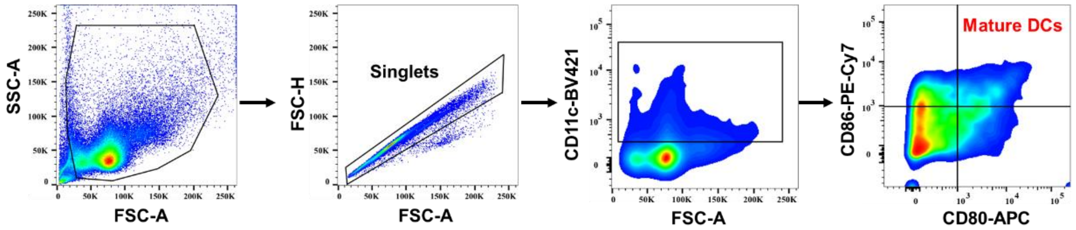

Figure S28. Gating manners for flow cytometry of mature DCs *in vitro* and *in vivo*.

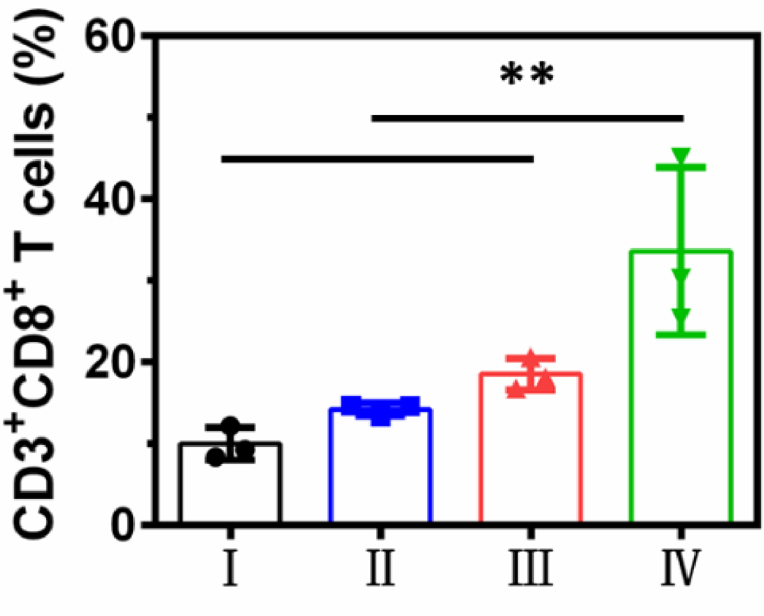

Figure S29. Percentage of tumor-infiltrating CD8+ (CD3+ CD8+) T cells in tumors isolated on day 5 after receiving indicated treatments. For each group: I, PBS; II, anti-PD-L1+X-ray; III, TZM+X-ray; IV, TZM + X-ray + anti-PD-L1. Data are presented as mean ± standard deviation, n = 3. Statistical analysis was performed using one-way ANOVA with Tukey's post-hoc test, $^{**}p < 0.01$.

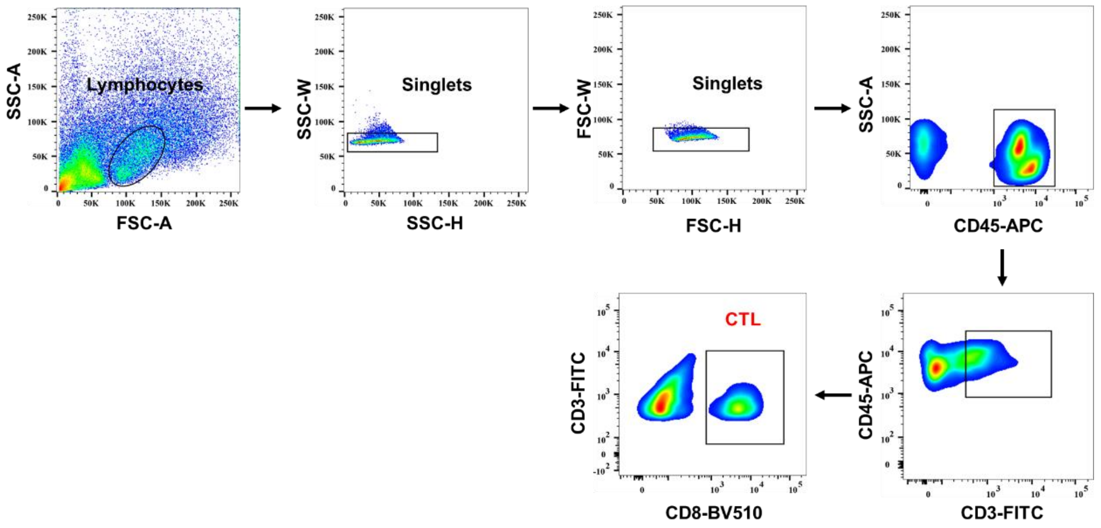

Figure S30. Gating manners for flow cytometry of $CD3^+$ and $CD8^+$ T cells.

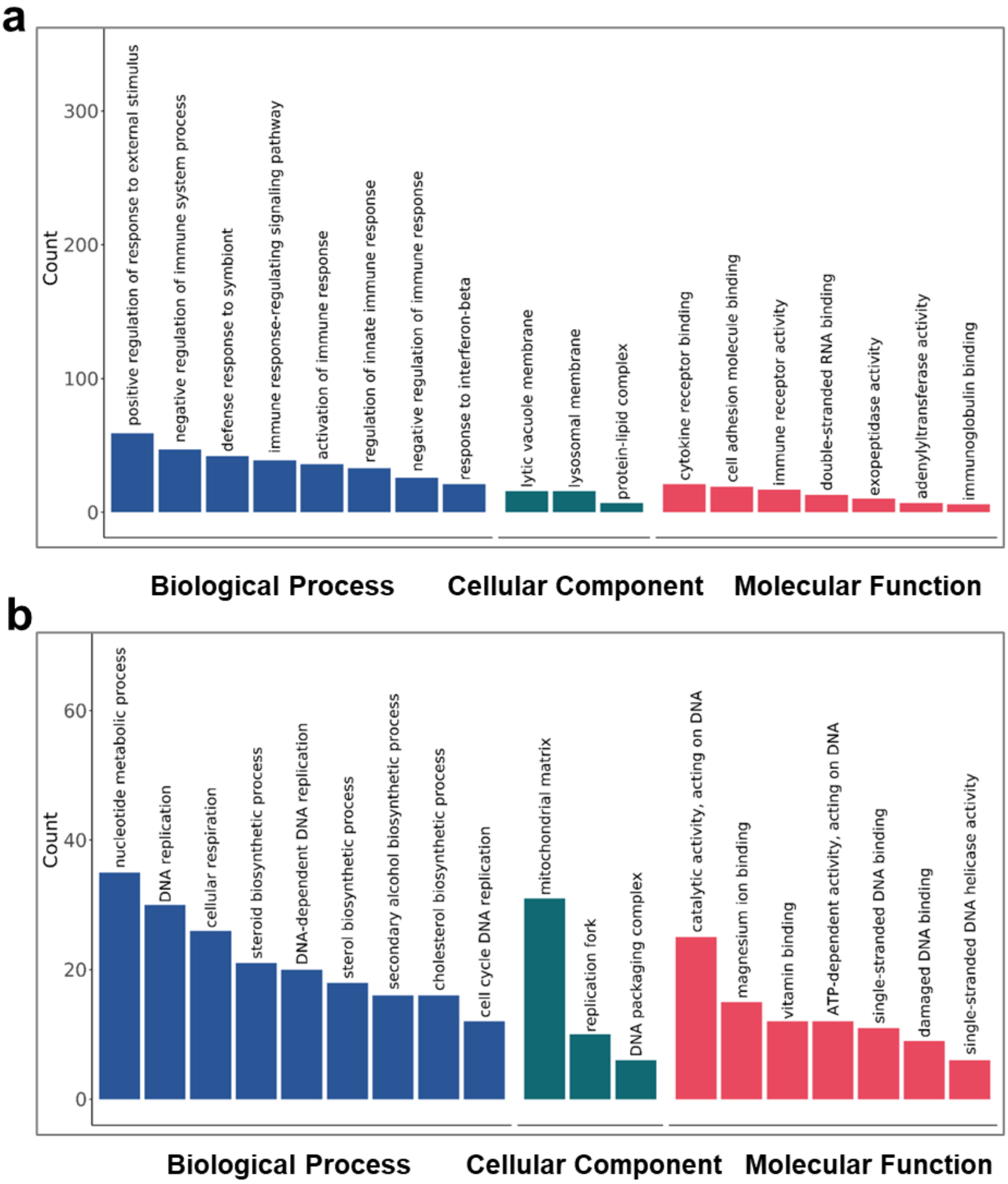

Figure S31. GO enrichment analysis of the gene functions of the (a) upregulated and (b) downregulated DEGs (TZM+X-ray vs. X-ray).

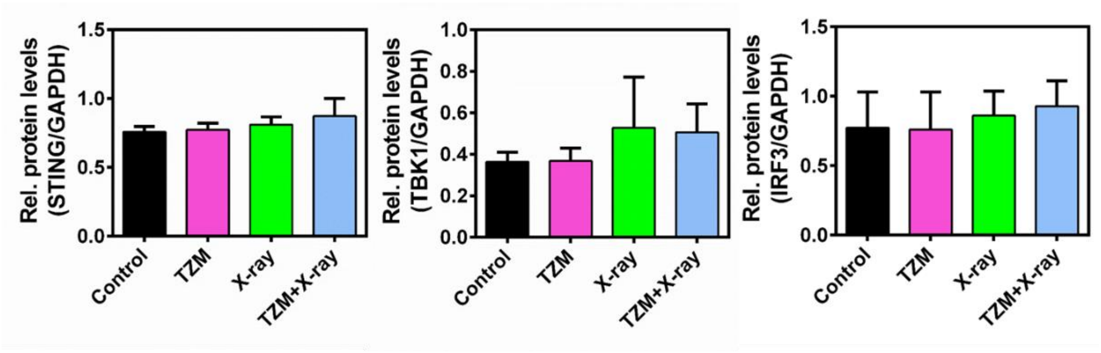

Figure S32. Semi-quantitative analysis of STING, TBK1, and IRF3 expression through western blot after different treatments. Data are presented as mean ± standard deviation, n = 3.

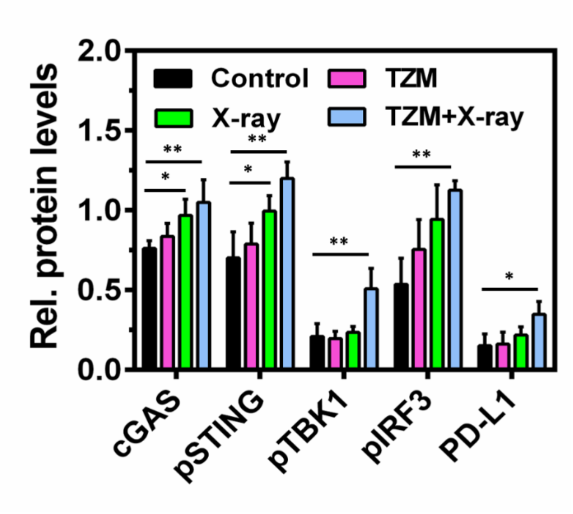

Figure S33. Semi-quantitative analysis of cGAS, pSTING, pTBK1, pIRF3, and PD-L1 expression through western blot after different treatments. Data are presented as mean ± standard deviation, n = 3. Statistical analysis was performed using one-way ANOVA with Tukey's post-hoc test, *$p < 0.05$ and **$p < 0.01$.

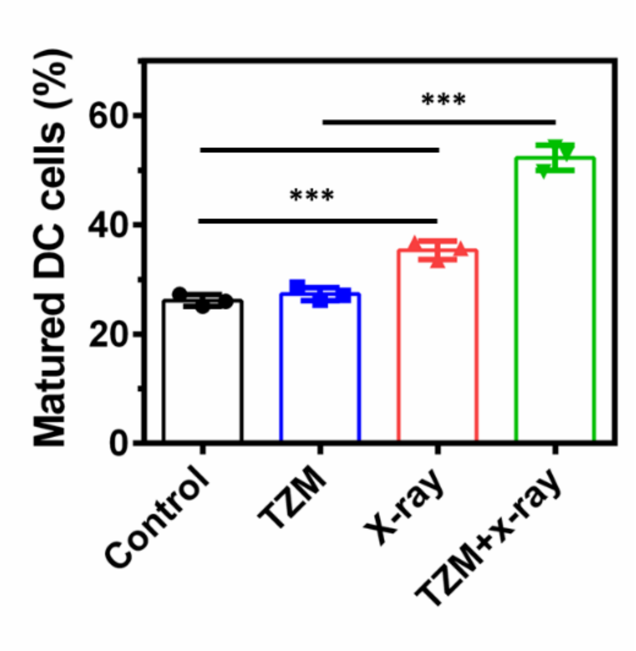

Figure S34. Percentage of mature DCs after different treatments. Data are presented as means ± standard deviations, n = 3. Statistical analysis was performed using one way ANOVA with Tukey's post-hoc test, ***p < 0.001.

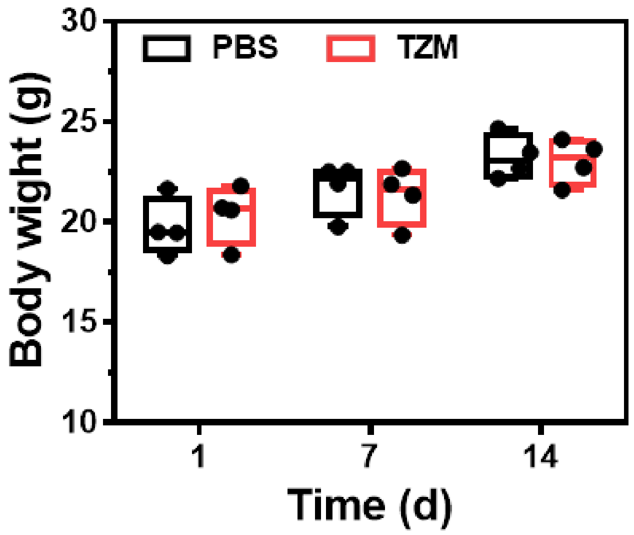

Figure S35. Body weight of mice after the intravenous injection of PBS and TZM. Data are presented as mean ± standard deviation, n = 4.

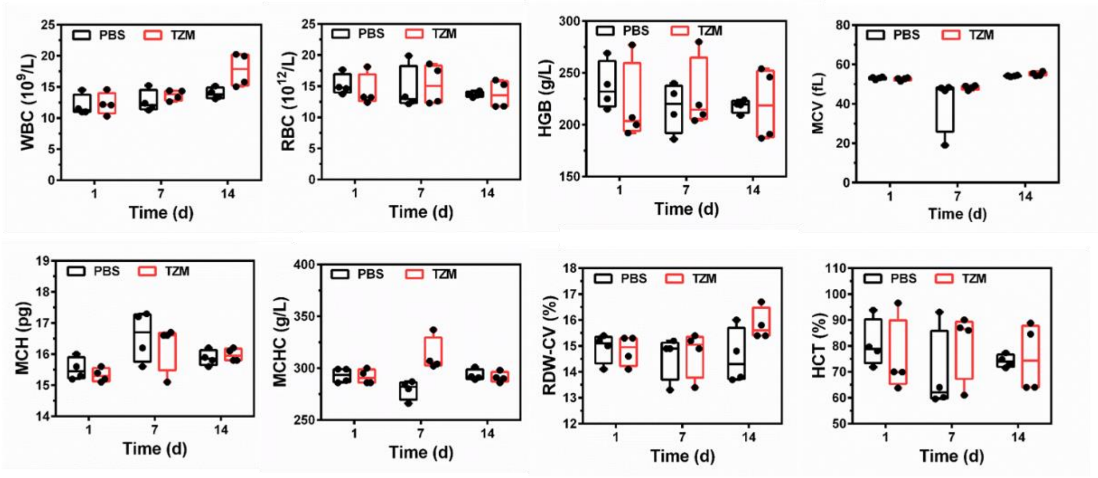

Figure S36. Blood routine test of mice treated with PBS or TZM. WBC, white blood cell; RBC, red blood cell; HGB, hemoglobin; MCV, mean corpuscular volume; MCH, mean corpuscular hemoglobin; MCHC, mean corpuscular hemoglobin concentration; RDW-CV, coefficient variation of RBC distribution width; HCT, hematocrit. Data are presented as mean ± standard deviation, n = 4.

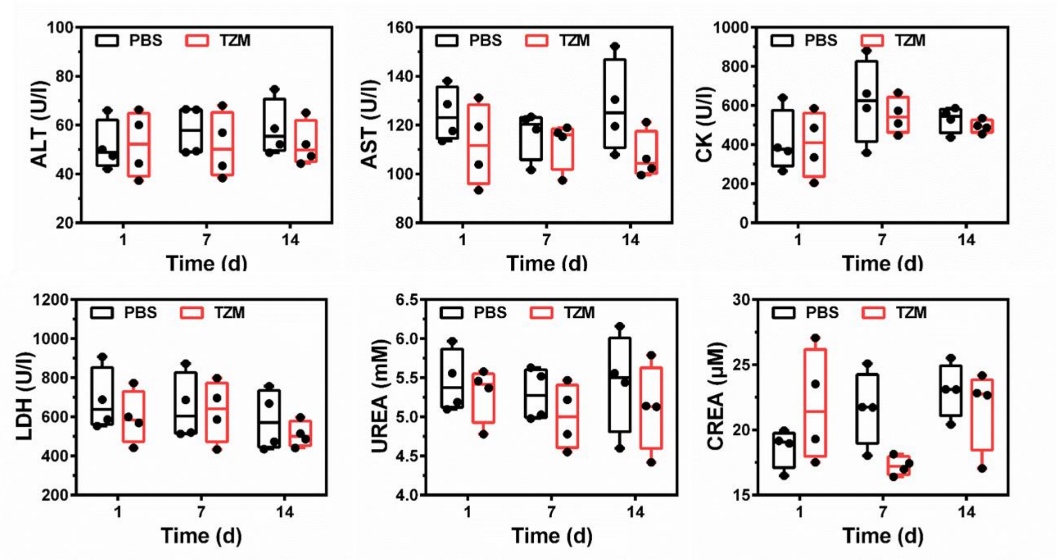

Figure S37. Blood biochemical analysis of mice treated with PBS or TZM. ALT, alanine aminotransferase; AST, aspartate transaminase; CK, creatine kinase; LDH, lactate dehydrogenase; blood urea nitrogen, UREA; CREA, creatinine. Data are presented as mean ± standard deviation, n = 4.

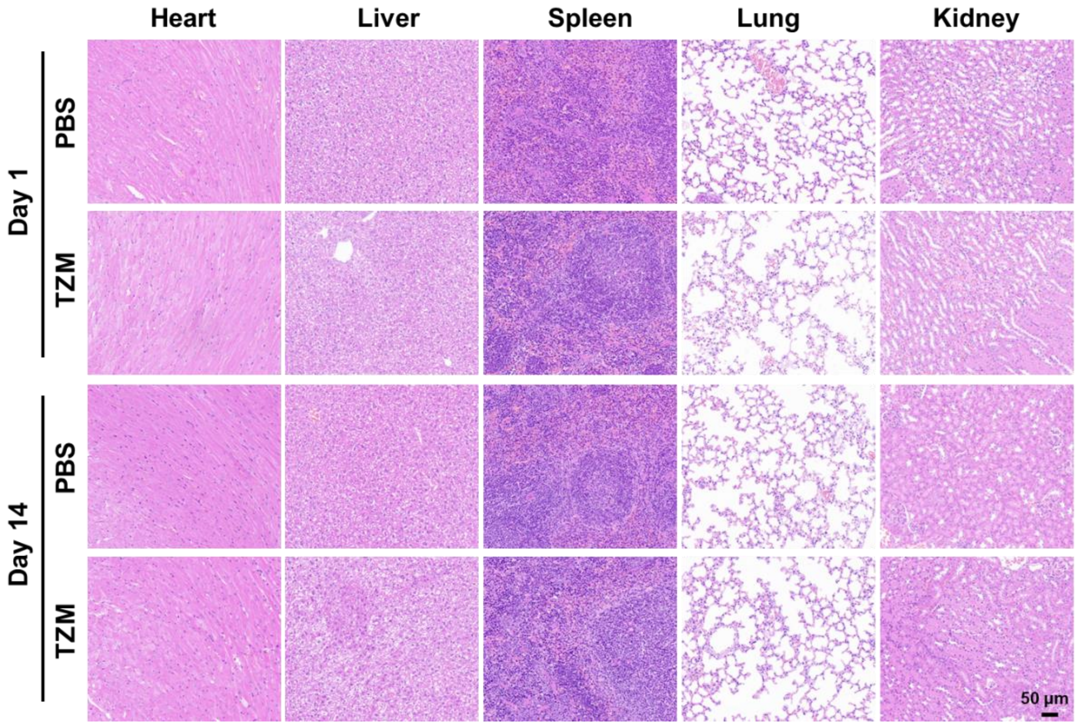

Figure S38. H&E staining of the major tissues (heart, liver, spleen, lung, and kidney) at days 1 and 14 after the intravenous injection of PBS or TZM.

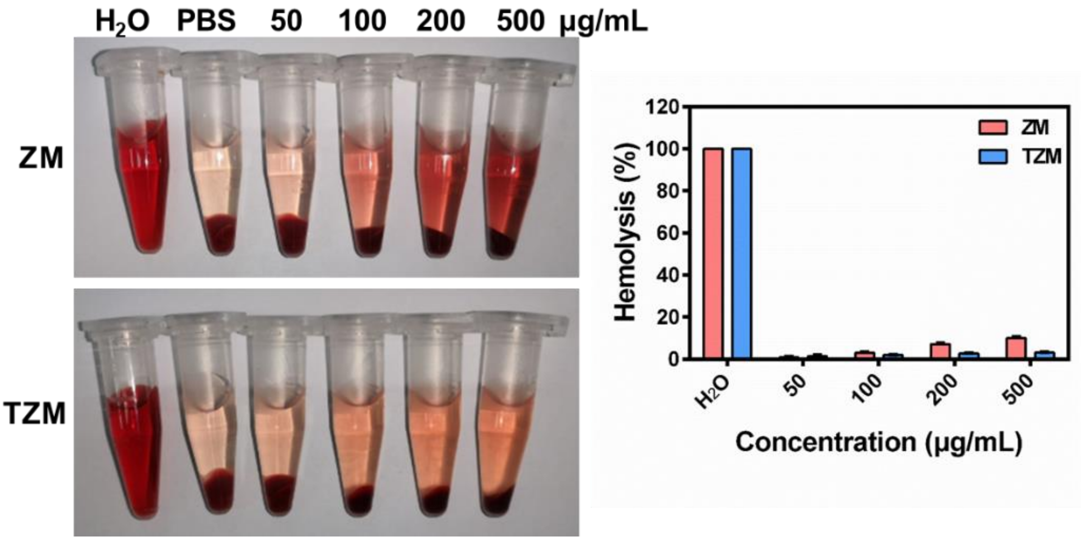

Figure S39. (a) Digital photographs of hemolysis of red blood cells (RBCs) incubated with various concentrations of ZM and TZM for 3 h. RBCs dispersed in D.I. water and PBS served as positive and negative control, respectively. (b) Calculated hemolysis ratios of RBCs in (a). Data are presented as mean ± standard deviation, n = 3.

Table S1. ICP-MS tests to determine the contents of Zr and Ta in TZM.

ZM solution (mL)	$TaCl_5$ (mg/mL)	Zr (ng/mL)	Mean±SD (ng/mL)	Ta (ng/mL)	Mean±SD (ng/mL)
10	2	199.2	184.8±14.0	121.1	139.3±43.9
10	2	183.9		107.4	
10	2	171.3		189.3	
10	5	195.6	187.7±8.1	574.0	540.7±29.5
10	5	188.0		517.8	
10	5	179.5		530.3	
10	10	190.0	170.4±17.8	1124.8	1171.2±105.3
10	10	155.4		1097.1	
10	10	165.8		1291.7	
10	20	102.7	97.8±12.1	1219.3	1201.6±72.0
10	20	106.7		1122.4	
10	20	84.0		1263.0	
